# Patient-clinician digital health interventions for the hip fracture population: a scoping review

**DOI:** 10.1186/s12913-023-09784-y

**Published:** 2023-10-02

**Authors:** Chantal Backman, Steve Papp, Anne Harley, Becky Skidmore, Maeghn Green, Soha Shah, Randa Berdusco, Stéphane Poitras, Paul E. Beaulé, Veronique French-Merkley

**Affiliations:** 1 School of Nursing, Faculty of Health Sciences, University of Ottawa, Ottawa Hospital Research Institute, Affiliate Investigator, Bruyère Research Institute, 451, Smyth Road, RGN 3239, Ottawa, ON K1H 8M5 Canada; 2 Faculty of Medicine, University of Ottawa, The Ottawa Hospital, Civic Campus, 1053 Carling Ave, Ottawa, ON K1Y 4E9 Canada; 3 Faculty of Medicine, University of Ottawa, 43 Bruyère St, Ottawa, ON K1N 5C8 Canada; 4 University of Ottawa, Ottawa, ON K1H 8M5 Canada; 5 The Ottawa Hospital, General Campus, 501 Smyth Rd, Ottawa, ON K1H 8L6 Canada; 6 Geriatric Rehabilitation, Bruyère Continuing Care, 43 Bruyère St, Ottawa, ON K1N 5C8 Canada; 7 Faculty of Medicine, University of Ottawa, The Ottawa Hospital, General Campus, 501 Smyth Rd, Ottawa, ON K1H 8L6 Canada; 8 School of Rehabilitation Sciences, Faculty of Health Sciences, University of Ottawa, 451, Smyth Road, Ottawa, ON K1H 8M5 Canada

**Keywords:** Digital health, Targeted patient/client communication, Hip fractures, Scoping review

## Abstract

**Introduction:**

Older adults with hip fracture often require extensive post-surgery care across multiple sectors, making follow-up care even more important to ensure an ideal recovery. With the increased adoption of technology, patient-clinician digital health interventions can potentially improve post-surgery outcomes of hip fracture patients by helping them and their caregivers better understand the various aspects of their care, post-hip fracture surgery. The purpose of this study was to examine the available literature on the impact of hip fracture-specific, patient-clinician digital health interventions on patient outcomes and health care delivery processes. We also aimed to identify the barriers and enablers to the uptake and implementation of these technologies and to provide strategies for improved use of these digital health interventions.

**Methods:**

We conducted a scoping review following the six stages of Arksey and O’Malley’s framework and following the PRISMA-ScR reporting format. Searches were conducted in five databases. In addition to hand searching for relevant studies from the references of all included studies, we also conducted a grey literature search to identify relevant primary studies. Screening of titles and abstracts as well as full texts were performed independently by two reviewers. Two reviewers also performed the data extraction of the included studies.

**Results:**

After screening 3,638 records, 20 articles met the criteria and 1 article was identified through hand searching. Various patient-clinician digital health interventions were described including telehealth /telerehabilitation programs (*n* = 6), care transition /follow-up interventions (*n* = 5), online resources (*n* = 2), and wearable devices /sensor monitoring (*n* = 1). Outcomes were varied and included functional status, gait/mobility, quality of life, psychological factors, satisfaction, survival/complications, caregiver outcomes, compliance, technology-user interactions, and feedback on the use of the digital health interventions. For clinicians, a key barrier to the use of the digital health interventions was the *acceptability of the technology*. However, the *usefulness of the digital health intervention* by clinicians was seen as both a barrier and an enabler. For patients and caregivers, all the themes were seen as both a barrier and an enabler depending on the study. These themes included: 1) *availability and access,* 2) *usability*, 3) *knowledge and skills*, 4) *acceptability*, and 5) *usefulness of the digital health intervention.*

**Conclusion:**

Many behavioural factors affect the use of patient-clinician digital health interventions. However, a specific attention should be focused on the acceptability of the technology by the clinicians to encourage uptake of the digital health interventions. The results of this scoping review can help to better understand the factors that may be targeted to increase the use of these technologies by clinicians, patients, and caregivers.

**Supplementary Information:**

The online version contains supplementary material available at 10.1186/s12913-023-09784-y.

## Introduction

Hip fractures are a significant health issue among older adults that require cohesive follow up care to ensure ideal recovery. Post-hip fracture surgery care is often provided across multiple health care sectors and typical follow-up care post-surgery can include pain control and management, osteoporosis assessment and treatment, fall risk prevention interventions, physical rehabilitation, assistive walking devices and/or home modifications, as well as follow-up visits with the orthopaedic surgeon and a primary care provider [1].

Digital health interventions have gained attention as potential tools to support patient-clinician interactions and improve outcomes for individuals with hip fractures [2]. With the increased accessibility to technology, patient-clinician digital health interventions are becoming a more viable option to help hip fracture patients and their caregivers as they navigate the modern health care system for their post-hip fracture surgery care [2].

In recent years, the inclusion of digital health technologies into healthcare processes has been identified as a global priority [3]. The term ‘Digital Health’ covers a variety of different tools including clinical decision support systems, electronic health record tools, patient-clinician communication applications, educational tools, and novel artificial intelligence (AI) algorithms [4]. The World Health Organization classifies patient-clinician digital health interventions as “targeted patient/client communication” technologies [5]. This type of technology intervention typically involves the use of various information and communication technologies to support the exchange of knowledge between clinicians and their patients regarding their care [5]. Providing technology-based interventions to patients and their caregivers can help improve healthcare delivery processes by better engaging them in managing their care and preventing hospital readmissions [2].

Recent reviews of digital health interventions for older adults with hip fracture found that interventions mainly supported physicians in improving their delivery of clinical care [6], with another meta-analysis of randomized control trials (*n* = 5) showing that nurse/physician voice telephone calls and educational videos interventions were two times more effective to prevent secondary fractures when compared to usual care (OR 2.13, CI 1.30–3.48) [7]. However, there is limited information on the specific components of patient-clinician digital health interventions (e.g., web-based applications, mobile applications, wearable devices, etc.) that are optimal to enhance patient engagement for this cohort. The purpose of this study was to examine the available literature on the impact of hip fracture-specific, patient-clinician digital health interventions on patient outcomes and health care delivery processes. We also aimed to identify the barriers and enablers to the uptake and implementation and to provide strategies for improved use of these digital health interventions.

## Methods

### Design and methodology

We conducted a scoping review following the six stages of Arksey and O’Malley’s framework [8] and followed the Preferred Reporting Items for Systematic Reviews and Meta-Analyses Statement for the Scoping Reviews (PRISMA-ScR) reporting format [9].

We used the Population-Concept-Context (PCC) acronym: P-Population (hip fracture patients 50 years of age or older who had surgical repair), C-concept (post-surgery care (e.g., pain control and management, mobilization, follow-up appointments) using any patient-clinician digital health interventions such as mobile technology, web-based applications, digital communication tools), C-context (care across various health care settings), design (all studies), Language (English or French), and year (all years).

Our protocol was registered in Open Science Framework (https://osf.io/w6a89)​​ [10] and published elsewhere [11].

### Patient and public involvement

No patients were involved.

- (1) Identifying the research question

The following research questions were identified for this review:

1. What is the impact of patient-clinician digital health interventions for older adults with hip fracture on patient outcomes and health care delivery processes?
2. What are the barriers and enablers to the use of patient-clinician digital health interventions for clinicians and patients with a hip fracture?
3. What strategies exist to improve the use of patient-clinician digital health interventions for hip fracture patients?

- (2) Identifying the research studies

An experienced medical information specialist developed and tested the search strategies through an iterative process in consultation with the review team. The MEDLINE strategy was peer-reviewed by another senior information specialist prior to execution using the PRESS Checklist [12].

Using the multifile option and deduplication tool available on the Ovid platform, we searched Ovid MEDLINE® ALL, Embase Classic + Embase, APA PsycInfo, and EBM Reviews (Cochrane Database of Systematic Reviews, CENTRAL and DARE). CINAHL was searched on Ebsco. All searches were performed on May 15, 2022.

The strategies utilized a combination of controlled vocabulary (e.g., “Hip Fractures”, “Telemedicine”, “Rehabilitation”) and keywords (e.g., “broken hip”, “digital health”, “post-surgical care”). Vocabulary and syntax were adjusted across the databases. There were no date or language limits but where possible, animal-only records were removed from the results. Records were downloaded and deduplicated using EndNote version 9.3.3 (Clarivate Analytics) and uploaded to Covidence [13]. We also performed citation searching of the included studies as well as a targeted grey literature search of clinical trial registries. Specific details regarding the search strategies appear in Appendix 1—search strategies.

- (3) Screening the studies

Studies were screened by two reviewers (CB, SH) using a two-step process. Specifically, the two reviewers independently screened titles and abstracts (level 1) according to the pre-determined eligibility criteria. For level 2 screening, the same two reviewers independently screened the full texts. Any disagreements were resolved by consensus.

- (4) Charting the data

Data were extracted using a piloted Microsoft Excel form. Two reviewers (CB, SH) extracted the data from the eligible studies. This included (1) general data (authors, year of publication, title, journal, country, purpose); (2) methodological data (study design, theoretical approach, type of participants, number of participants, description of the patient-clinician digital health intervention, data analysis); (3) study results/outcomes (patient outcomes, health care delivery processes; and (4) barriers and enablers. We used the Intervention Description and Replication (TIDieR) checklist to extract data about the interventions reported in the included studies [14]. No risk of bias assessment was performed in this scoping review.

- (5) Summarizing and collating the data

The general, methodological and results/outcomes of the included studies were analyzed using narrative synthesis to summarise and explain the findings. The data was grouped by interventions, and by outcomes (patient outcomes, health care delivery processes).

The barriers and enablers were analyzed using a qualitative descriptive approach. The Theoretical Domain Framework (TDF) [15, 16] guided the qualitative analysis of the barriers and enablers. Two reviewers (CB, SH) independently grouped the data extracted from the included studies into themes and coded each theme as a barrier or an enabler. The themes were then mapped to each of the TDF domains. For each barrier and enabler, frequency and percentage were reported to identify the top domains. Any disagreements were discussed and resolved by consensus. We identified behavioural change techniques [17] that could address the barriers and enablers identified in the review to help guide the uptake of future patient-clinician digital health interventions for older adults with hip fracture transitioning from hospital to rehabilitation to home. Examples of behavioural change techniques included restructuring the physical and social environments, practicing and giving feedback, providing incentives, identifying social and environmental consequences, utilizing verbal persuasion to boost self-efficacy, explaining pros and cons, providing prompts/cues, and providing appropriate social support.

- (6) Consulting with stakeholders

As the last stage of the Arksey and O’Malley’s framework [8], we consulted with a small number of clinical experts on our team (MG, SP, PB, AH, VFM) and a digital health developer (NexJHealth, nexjhealth.com). No additional patient-clinician digital health interventions were identified. Feedback provided about the review findings was incorporated into the discussion.

## Results

### Study selection

**Fig. 1:**
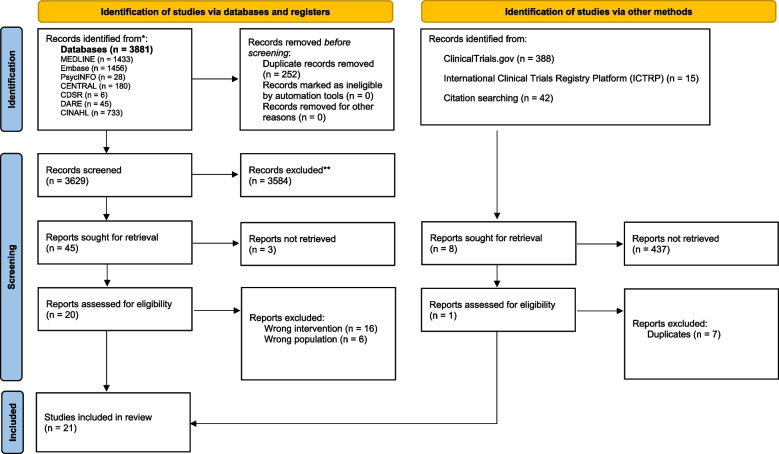
PRISMA diagram

A total of 3,638 records were retrieved from the search, of which 9 were duplicates, and 3,584 were excluded at the title and abstract stage. This resulted in a total of 45 studies assessed for full-text eligibility. A total of 25 studies were excluded for wrong intervention (*n* = 16), wrong patient population (*n* = 6), and full-text not available (*n* = 3). The reasons for exclusion are noted in Appendix 2—list of excluded studies. In addition, a targeted grey literature search of ClinicalTrials.gov (*n* = 388), International Clinical Trials Registry Platform (ICTRP) (*n* = 15) and citation searching (*n* = 42) was performed. Twenty articles from the database searches met our inclusion criteria as well as one additional recent article (published in 2022) was identified through the citation searching. Thus, a total of 21 articles [18–38] were included in this review (Fig. 1—PRISMA diagram).

### Characteristics of included studies

The included articles were conducted in Spain (*n* = 4) [18–21], United States (*n* = 4) [22–25], Australia (*n* = 3) [26–28], China (*n* = 3) [29–31], Denmark (*n* = 2) [32, 33], Netherlands (*n* = 2) [34, 35], Canada (*n* = 1) [36], South Korea (*n* = 1) [37], and Israel (*n* = 1) [38].

Study designs included randomized controlled trial (*n* = 4) [30, 31, 35, 38], non-randomized trial (*n* = 2) [20, 21], quasi-experimental (*n* = 2) [22, 29], observational/feasibility (*n* = 5) [24, 28, 34, 36, 37]. In addition, there were qualitative (*n* = 6) [18, 19, 23, 25, 26, 33], participatory/co-design (*n* = 1) [32] and mixed methods (*n* = 1) [27] studies. Majority of studies (81%, *n* = 17) were published after 2017.

Studies took place in hospital only (*n* = 2) [27, 37], in both hospital and home (*n* = 6) [23–25, 30, 32, 33], in home only (*n* = 10) [18–22, 28, 29, 31, 34, 38], in rehabilitation and home (*n* = 2) [35, 36], or in various settings (*n* = 1) [26]. Further details are available in Table 1.

**Table 1:** Characteristics of included studies (*n* = 21)

Lead author (year)	Country	Purpose / Study objectives	Study Design	Setting	Participants (N)	Name of Digital Health Intervention	Digital health tools	Post-surgery care	Users of the digital health tools	Function of the digital health tools
Ariza-Vega 2021a [18]	Spain	To describe family caregivers experience with the @ctivehip telerehab program	Qualitative study	Home	21 caregivers	@ctivehip	Telerehabilitation	Rehabilitation exercises (Physical Therapy, Occupational Therapy)	Patients, caregivers, occupational therapists (OT), physiotherapists (PT)	Management continuity Deliver pre-recorded occupational therapy and physiotherapy exercises through online platform, videoconferencing with clinician
Ariza-Vega 2021b [19]	Spain	To explore family caregivers' perspectives of the recovery process of older adults with hip fracture and describe experiences from caregivers who: (1) used the online intervention, or (2) received home-based care provided by the Andalusian Public Health Care System	Qualitative study	Home	44 caregivers	@ctivehip	Telerehabilitation	Rehabilitation exercises (Physical Therapy, Occupational Therapy)	Patients, caregivers, occupational therapists (OT), physiotherapists (PT)	Management continuity Deliver pre-recorded occupational therapy and physiotherapy exercises through online platform, videoconferencing with clinician
Ortiz-Pina 2021 [20]	Spain	To design a home-based multidisciplinary tele-rehabilitation protocol for patients with hip fracture, and to compare this protocol versus the home-based usual outpatient rehabilitation protocol	Single-blinded, non-randomized clinical trial	Home	62 patients	@ctivehip	Telerehabilitation	Rehabilitation exercises (Physical Therapy, Occupational Therapy)	Patients, caregivers, occupational therapists (OT), physiotherapists (PT)	Management continuity Deliver pre-recorded occupational therapy and physiotherapy exercises through online platform, videoconferencing with clinician
Mora-Traverso 2022 [21]	Spain	To test the effects of the @ctivehip telerehabilitation program on the quality of life, psychological factors and fitness level of patients who had suffered a hip fracture	Non-randomized clinical trial	Home	71 patients	@ctivehip	Telerehabilitation	Rehabilitation exercises (Physical Therapy, Occupational Therapy)	Patients, caregivers, occupational therapists (OT), physiotherapists (PT)	Management continuity Deliver pre-recorded occupational therapy and physiotherapy exercises through online platform, videoconferencing with clinician
Bedra 2015 [22]	USA	To assess impact of home-based telerehabilitation of community dwelling older adults in post-acute phase of recovery after hip fracture on mobility, psycho-behavioral factors, quality of life, and satisfaction with care; and to estimate acceptance of the telerehabilitation system and adherence to the exercise program	Quasi-experimental pre/post design	Home	10 patients	Hip Hat System	Telerehabilitation	Rehabilitation exercises (Physical Therapy), Post-hip fracture rehab education module	Patients, physiotherapists	Management and Informational continuity Provide individualized exercise programs and educational module
Nahm 2012a [23]	USA	To discuss our recruitment process and the lessons learned	Qualitative study	Hospital and Home	36 dyads (1 patient-1 caregiver)	Online hip fracture caregiver resource center (OHRC)	Web-based resources	Coping with the CG role, care needs during hospitalization, care needs in rehab, care needs at discharge to home or facility, prevention of future fractures	Caregivers, nurses	Informational continuity The OHRC was developed to provide CGs with the anticipatory knowledge and skills that they need to manage upcoming caregiving situations and cope with the potential challenges
Nahm 2012b [24]	USA	To develop a theory-based online hip fracture caregiver (CG) resource center (OHRC) program for caregivers and to conduct a feasibility study to test the OHRC for a future randomized controlled trial	Feasibility study	Hospital and Home	36 dyads (1 patient-1 caregiver)	Online hip fracture caregiver resource center (OHRC)	Web-based resources	Coping with the CG role, care needs during hospitalization, care needs in rehab, care needs at discharge to home or facility, prevention of future fractures	Caregivers, nurses	Informational continuity The OHRC was developed to provide CGs with the anticipatory knowledge and skills that they need to manage upcoming caregiving situations and cope with the potential challenges
Nahm 2013 [25]	USA	To explore the caregivers’ experiences in taking care of their care recipients while they were using the OHRC resource center over the 8-week period through the analysis of discussion board postings	Qualitative study	Hospital and Home	36 dyads (1 patient-1 caregiver)	Online hip fracture caregiver resource center (OHRC)	Web-based resources	Coping with the CG role, care needs during hospitalization, care needs in rehab, care needs at discharge to home or facility, prevention of future fractures	Caregivers, nurses	Informational continuity The OHRC was developed to provide CGs with the anticipatory knowledge and skills that they need to manage upcoming caregiving situations and cope with the potential challenges
Yadav 2021a [26]	Australia	To understand stakeholders' perspectives on the development of a digital health-enabled model of care for fragility hip fractures and to map out factors that could influence the design and implementation of such a model	Descriptive qualitative	Multiple health settings	24 clinicians	Digital patient health hub	Web-based resources	Personalized patient education	Patients, caregivers and clinicians	Management and Informational continuity The digital health hub was designed to improve education, service integration, data exchange and engagement of all stakeholders including patients and clinicians
Yadav 2021b [27]	Australia	To understand the perspectives of older patients with hip fracture and their family members and residential aged caregivers on the feasibility of developing a model of care using a personalized digital health hub	Mixed methods	Hospital	55 patients, 13 family members, 32 facility caregivers	Digital patient health hub (Not developed at time of study)	Web-based resources	Pain management, medication management, rehabilitation exercises, pressure ulcer prevention, care needs during hospitalization, care needs at discharge follow-up community appointments	Patients, caregivers, and residential aged care staff	Management and Informational continuity This proposed web-based health information portal, or a website, is intended for patients who can access all relevant information about their hip fractures
Morris 2021 [28]	Australia	To evaluate the provision of tele-rehabilitation to older people with recent hip or pelvic fractures as they are discharged from hospital and enter a home rehabilitation service	Prospective observational study	Home	52 patients	Telerehabilitation (TR)	Telerehabilitation	Rehabilitation exercises (Physical Therapy, Occupational Therapy), fall prevention, medication management	Patients, nurses, allied health including physiotherapy	Management and Informational continuity Deliver remote prescription of exercises with demonstration videos, and videoconferencing with clinicians
Gao 2021 [29]	China	To explore the role of chat software in rehabilitation guidance for hip fracture patients during COVID-19	Quasi-experimental study	Home	80 patients	WeChat Group	Chat Software	Follow-up orthopaedic recommendations	Patients, physicians	Management and Informational continuity Chat software for patient-physician communication, monitoring patient status and educating patients
Li 2022 [30]	China	To investigate the effects of a home-based occupational therapy telerehabilitation (TR) via smartphone in enhancing functional and motor performance and fall efficacy for outpatients receiving day hospital rehabilitation after hip fracture surgery in Hong Kong	Randomized controlled trial	Hospital and Home	31 patients	Caspar Health e-system	Telerehabilitation	Rehabilitation exercises (Occupational Therapy)	Patients, occupational therapist	Management and Informational Continuity Deliver exercise program and facilitate communication between patient-clinician
Cheng 2022 [31]	China	To test the effectiveness of a mobile app in delivering home-based rehabilitation program for improving functional outcomes and reducing caregiver stress with enhancing adherence among the elderly patients with hip fracture	Randomized controlled trial	Home	50 patients	Mobile App	Telerehabilitation	Rehabilitation exercises (Physiotherapy), general knowledge post-hip fracture management, community resources, caregiver skill and information	Patients, caregivers, physiotherapists	Management and Informational continuity The mobile app was developed to facilitate the implementation of a home-based rehabilitation program. Participants could use this mobile app to follow home-based exercises prescribed by their physiotherapists, track their exercise progress and obtain relevant information about hip fracture re-habilitation
Jensen 2018 [32]	Denmark	To investigate whether a user-driven approach in a participatory design can provide a solution to bridge the gap between what the healthcare system provides and what patients need after being treated for a hip fracture, during a short period of hospitalisation	Participatory co-design	Hospital and Home	Phase 1: 10 patients and 4 caregivers Phase 2: 3 patients	My Hip Fracture Journey	Mobile application	Self-care Post-hip fracture rehab education	Patients, caregivers, unspecified clinical staff	Management and Informational continuity Support for clinical staff in the daily provision of oral and written information and education of patients in accordance with local clinical guidelines
Jensen 2019 [33]	Denmark	To investigate whether a tele-health solution, an "app" presented on a tablet, can assist patients in their recovery following a hip fracture and accommodate individual learning and health literacy needs to support them in self-care and empowerment	Qualitative study	Hospital and Home	20 patients	My Hip Fracture Journey	Mobile application	Self-care Post-hip fracture rehab education	Patients, caregivers, unspecified clinical staff	Management and Informational continuity Support for clinical staff in the daily provision of oral and written information and education of patients in accordance with local clinical guidelines
Geerds 2020 [34]	Netherlands	To investigate the real-world use of a mobile app for monitoring postoperative functional recovery after hip fracture	Feasibility study	Home	110 patients or their caregivers	Mobile App for post-op monitoring	Mobile application	Monitoring post-op functional recovery	Patients, caregivers, nurses	Management Continuity Mobile app for monitoring postoperative functional recovery after discharge
Pol 2019 [35]	Netherlands	To test the effects of an intervention involving sensor monitoring informed occupational therapy on top of a cognitive behavioral treatment (CBT) based coaching program on patient-reported daily functioning in older patients after hip fracture	Three-armed randomized stepped wedge trial	Rehabilitation and Home	240 patients	Sensor monitoring system	Wearable device and motion sensors	Rehabilitation (Occupational Therapy), Fall Management	Patients, occupational therapists	Management Continuity Sensor monitoring informed occupational therapy on top of cognitive-behavioral treatment (CBT) based coaching program
Backman 2020 [36]	Canada	To develop and test the feasibility of a novel web-based application called MyPath to Home that can be used to manage the personalized needs of geriatric rehabilitation patients during their transition from the hospital to home	Feasibility study	Rehabilitation and Home	34 patients, 19 caregivers, 20 clinicians	MyPath to Home	Web-based application	Pain management, fall prevention, medication management, information about geriatric rehabilitation, follow-up with clinicians, community resources, equipment needs	Patients, caregivers, clinicians (physiotherapists, social workers, occupational therapists, physicians, nurses)	Management and Informational Continuity MyPath to Home web-based application was developed to serve as a digital care transition record for geriatric patients with hip fractures
Ko 2021 [37]	South Korea	To develop rehabilitation instructions in the form of a mobile application for the physical recovery of older adults after hip fracture surgery	Feasibility study	Hospital	9 clinicians	Rehabilitation instructions after hip fracture surgery	Mobile application	Rehabilitation exercises (Occupational therapy and Physiotherapy, activities of daily living, pain management, nutrition management, fall prevention, follow-ups	Patients, clinicians (clinical instructors, orthopedic surgeon, head nurses, orthopedic nurses)	Management and Informational continuity Mobile application for rehabilitation instructions after hip fracture
Kalron 2018 [38]	Israel	To examine the effects of a 6-week telerehabilitation program on the mobility of people following hip surgery and compare the results with those who only received an exercise booklet	Feasibility pilot randomized control study	Home	40 patients	Telerehabilitation program based on a video platform for therapy software program	Telerehabilitation	Rehabilitation exercises (Physiotherapy)	Patients	Management continuity Deliver pre-recorded physiotherapy exercises through online platform

### Patient-clinician digital health interventions

Several types of patient-clinician digital health interventions to facilitate patient-clinician interactions were identified. These included:

- 1) Telehealth /telerehabilitation programs (*n* = 6): @ctivehip [18–21], Hip Hat System [22], Telerehab (TR) [28], CASPAR Health e-system [30], Telerehabilitation program based on a video platform for therapy software program [38], and Mobile App [31]. These programs allow for virtual consultations and enabling timely follow-ups. Real-time video conferencing facilitates direct communication between patients and clinicians, allowing for the assessment of progress, medication management, and addressing concerns or questions.
- 2) Care transition /follow-up interventions (*n* = 5): My Hip Fracture Journey [32, 33], Mobile App for post-op monitoring [34], MyPath to Home [36], WeChat Group [29], Rehabilitation instructions after hip fracture [37]. Mobile applications and web-based applications can deliver personalized reminders and notifications regarding medication schedules, appointments, and rehabilitation exercises, fostering adherence and continuity of care.
- 3) Web-based resources (*n* = 2): Online hip fracture caregiver resource center [23–25], and Digital patient health hub [26, 27]. These educational resources can be in the form of videos, and educational modules, providing patients with information about their condition, treatment options, rehabilitation exercises, and self-care practices.
- 4) Wearable devices /sensor monitoring (*n* = 1) [35]. Remote monitoring devices enable clinicians to remotely assess patients' vital signs, activity levels, and adherence to treatment protocols.

Interventions focused on the post-surgery care, including rehabilitation exercises (*n* = 13), follow-up and management (*n* = 9), post-hip fracture education and self-care (*n* = 4), and caregiver needs (*n* = 3).

Digital health tools used were primarily tele-rehabilitation software (*n* = 9), web-based applications (*n* = 6), mobile applications (*n* = 4), wearable devices (*n* = 1), and chat software (*n* = 1).

### Functions of the digital health tools

The digital health tools’ functions were grouped according to the 3 categories of the continuity of care framework [39]. The continuity of care framework is defined as *“the degree to which a series of discrete healthcare events is experienced as coherent and connected and consistent with the patient's medical needs and personal context”* ([39], p.1221). The framework consists of these 3 categories: 1) informational continuity (use of information available to all clinicians to make care appropriate for each patient), 2) management continuity (consistent and coherent coordination between all involved in the care that is responsive to a patient’s needs), and 3) relational continuity (a trusting therapeutic relationship between a patient and at least one caring clinician) as key components of high-quality care. Most studies were focused on management continuity (*n* = 7) or informational continuity (*n* = 3) only. A total of 11 studies focused on both management and informational continuity. Details of the interventions are found in Table 2.

**Table 2:** Description of digital health interventions (following TIDierR checklist) [14]

Lead author (year)	Brief name of digital health intervention	Why?	What?	Who provided?	How?	Where?	When?	How much?	Tailoring?	How well?
**Telerehabilitation programs (defined as the delivery of rehabilitation using telecommunication technologies (*n*** **T= 6)**										
Ariza-Vega 2021a, Ariza-Vega 2021b, Ortiz-Pina 2021, Mora-Traverso 2022 [18–21]	@ctivehip	No theoretical framework provided	The @ctivehip intervention consisted of: (i) web-based information to increase family caregivers’ knowledge and skill development; (ii) a supported exercise and ADL program for older adults (delivered by the family caregiver); (iii) a specific section on family caregivers’ health; and (iv) an option for family caregivers to video conference with health professionals	Occupational therapist and physiotherapist	Web-based	Home	12 weeks post-discharge	5 online-based sessions per week each lasting 50–60 min	Four levels (Beginners, Moderate, Advanced 1, and Advanced 2)	Ariza-Vega 2021a: At the follow-up call, 3 caregivers did not answer the telephone after several attempts, and 4 caregivers withdrew from the study. Thus, in total, 44 caregivers were interviewed, representing 21 older adults with hip fracture from the intervention group and 23 older adults with hip fracture from the control group Ariza-Vega 2021b: Ten of twenty-one caregivers completed the program as intended (high fidelity at 12 weeks), and an additional six participants completed 8 weeks or more of the program (76% in total). Half of the caregivers (10/21; 48%) stated their older family member completed the program, and then continued doing the exercises for a few more months. However, the remaining caregivers reported their family member stopped doing the exercises before the end of the 12 weeks. Most family caregivers (20/21; 95%) expressed 12 weeks was long enough to learn the program, or they believed their family member did not require rehabilitation beyond 12 weeks Ortiz-Pina 2021: We observed variation for adherence to the tele-rehabilitation program, but in general, it decreased over time. In our study, only 15% of patients completed the full program (50–60 sessions), but 22 patients (63%) completed > 20 sessions. Mora-Traverso 2022: The adherence was 17% (n = 6) to the full @ctivehip rehabilitation program (50–60 sessions), 69% (n = 24) to at least 20 sessions and 89% (n = 31) to at least 10 sessions
Bedra 2015 [22]	Hip Hat System	Based on social cognitive theory	Home Automated Telemanagement (HAT) system including home unit (HU), HAT server and clinician unit, any web-enabled device HAT system was used to support an individualized exercise program (home unit guides patients, patient information reported back to physiotherapist) and a self-paced multimedia education module	Physiotherapist	Web-based	Home	30 days	30 sessions, 1 h/daily	The patient settings were individualized and were adjusted by the physiotherapist at the HAT website based on patient performance	Overall, 14 patients with confirmed diagnosis of the hip fracture were recruited to test the telerehabilitation system at their homes. One patient withdrew from the study and three patients moved out of town. Adherence to the exercise regimen was assessed using real-time exercise logs. Adherence to Exercises per Day over a 30 day monitoring: 89% Adherence to Sessions per day over a 30 day monitoring: 88% Adherence to Exercises per session per day over a 30 day monitoring: 87% Adherence to number of Sets per exercise per day over a 30 day monitoring: 97% Adherence to number of repetitions per set per exercise per session per day over a 30 day monitoring: 91%
Morris 2021 [28]	Telerehabilitation (TR)	No theoretical famework provided	Patients who received telerehabilitation are loaned a 4G enabled tablet on an adjustable stand. The tablet is configured with commercially available apps and a videoconferencing platform that can be used to provide all or some of the rehabilitation interventions. The exercise app allows the remote prescription of standard exercises accompanied by a demonstration video and the ability for clinicians to track adherence. Clinicians introduced the tablet during their first visit to the patient and provided basic training to the patient and their carer. A simple instruction booklet acts as a reminder for tasks such as turning the tablet on and off, opening apps and how to participate in a video call. Clinicians, supported by an IT professional, then used clinic-based VC equipment including desk top and wall mounted screens, cameras, microphones, and headsets. Remote access to tablet-based apps via a mobile device manager was available	Nurses, Allied Health including physiotherapy	Web-based	Home	Average 12.09 ± 3.62 days	TR substituted an average of 3 home visits for virtual visits	Together, clinicians and patients decide when and how frequently TR interventions are provided based on rehabilitation goals and progress	Of those, 35 (67%) patients were considered suitable for TR and agreed to receive their rehabilitation services by using TR (TR group). The remainder 17 (33%) HRS patients did not receive TR (nTR group). Of those in the nTR group, 6 patients lived in residential care, 2 were readmitted within 48 h, and 6 were considered by the therapists as unsuitable for TR due to hearing, vision or language deficits. An additional 3 people refused TR. Reasons for refusal were a dislike of new technology, a preference for face-to-face home visits only and feeling overwhelmed on discharge from hospital
Li 2022 [30]	Caspar Health e-system	No theroretical framework provided	Telerehabilitation was delivered through the Caspar Health e-system (CASPAR Health, Berlin, Germany), a German designed Internet system for desktop and a mobile app for both iOS and Android smartphones which enables patients to interact directly with and seek advice from the hospital or to do exercise anywhere according to the therapists’ treatment plan through digital communication. (1) Therapists set a tailormade TR programme for each patient through the e-system calendar, and data, such as exercise videos and frequency, are transferred to the patient’s mobile phone or tablet through the Caspar Health App. (2) The patient performs the home-based training using the videos, pictures and written and verbal instructions shown on the app, with or without assistance from their caregivers. (3) After practice, the patient uploads their training video or verbal feedback to the therapists so that the therapists can update the home programme according to the patient’s progress. The Caspar Health e-system also allows therapists to review patients’ attendance records and communicate with them if needed	Occupational Therapists	Web-based	Hospital and Home	3-week period intervention, post-intervention follow-up at 6 weeks	Not described	The contents of the home programme in both groups were tailor-made according to the needs of each case by occupational therapists who were not blinded to the treatment	Thirty-one patients were successfully recruited between June 2018 and May 2019. We identified several reasons for patients refusing to participate in the study: problems related to the procedures of the study (difficulty understanding the consent form and using the mobile app); fear of over-exercising apart from attending the standardised treatment in the day hospital; feeling overwhelmed in adapting to the standardised treatment in the day hospital; feeling fatigued after the study intake; and a feeling of uncertainty about joining the research. Eventually, 15 patients were allocated to the experimental group, and 16 patients were assigned to the control group. All patients completed the training programme, and 30 of them attended the follow-up session. One patient in the control group did not attend the follow-up session because of readmission to hospital A high adherence rate in terms of completing 90% of the home programme was found for both the experimental group (87%) and the control group (86%). Two patients in the experimental group only completed 50% of the home programme due to technical problems in using the app in the initial stage of the study. Two patients in the control group did not commit to the majority of the home programme due to low motivation and readmission to hospital, respectively
Kalron 2018 [38]	Telerehabilitation program based on a video platform for therapy software program	No theoretical framework provided	After an initial examination at the hospital, the therapist recommended an exercise program by selecting specific exercises according to the patient’s physical ability and in accordance with the rehabilitation goals. For the present study, only exercises relating to movement, strength of the lower limbs, and balance performance were included. The software allowed the therapist to adjust the number of repetitions and performance pace for each exercise. The therapist received on request, a report from the dedicated software, as to whether the patient performed the exercise program together with information relating to each exercise The software includes short video clips of common rehabilitation exercises (e.g. squats, lunges, heel rises, etc.) and an audio clip describing the different phases of the exercise and a depiction of correct versus incorrect performances	Physiotherapist	Web-based	Home	6 consecutive weeks, follow-up 4 weeks post-intervention	18 sessions, 3 sessions/week, and 40–50 min/session	Following each session, the patient was asked for feedback as to the difficulty of the exercises (e.g. easy, hard, and very hard) who was then sent to the therapist by the web site. According to the patient’s feedback, the therapist would readjust or change the program	Five participants from the telerehabilitation group and three from the control group withdrew from the program within the first 2 weeks owing to difficulties in arriving at the evaluation sessions/sickness/and need to return to work In terms of adherence, according to the self-report diary, 10 (out of 15) participants in the telerehabilitation group performed at least 15 training sessions, three performed between 10 and 14 sessions, and two performed up to 10 training drills. As for the control group, seven (out of 17) performed at least 15 training sessions, two between 10 and 14 sessions, and eight performed up to 10 training drills
Cheng 2022 [31]	Mobile App	No theoretical framework provided	A briefing session was arranged for all participants with their caregivers before hospital discharge The features of the app include: the exercise program, progress summary, push reminders, rehab knowledge, caregiver skills videos, support information The home-based rehabilitation program for hip fracture patients involved a combination of training focused on strength, coordination and functional movements of geriatric hip fracture patients	Physiotherapists	Web-based	Home	Follow-up 6 months post discharge	1x/daily for 20–30 min	Prescribed exercises based on assessments and progression of exercises monitored by weekly home visits	Eleven participants withdrew from the study, with eight participants refusing home visits, one participant having deterioration of medical condition and two participants being unable to contact
**Care transition/follow-up interventions (*n* = 5)**										
Jensen 2018, Jensen 2019 [32, 33]	My Hip Fracture Journey	No theoretical frameowrk provide	Patients participating in the test phase were given both oral and written information concerning the hip fracture treatment and according to local guidelines—and the tablet The app contained four main features: 1. pictographs, 2. video clips, 3. illustrated exercises, and 4. written information. Information on typical treatment pathway, video clips provided narratives from other patients, Information or education concerning the LOS and rehabilitation, pre-recorded exercise videos, FAQs	Clinical staff (discipline unspecified)	Mobile app	Hospital and Home	Not described	Not described	The ‘‘My Hip Fracture Journey’’ app aimed to accommodate individual needs and learning styles	A total of 25 patients who met the inclusion criteria were included in the test period. Five participants dropped out due to changing their mind about participation The test phase ended in May 2018 with 20 patients having tested the app using the tablet in hospital and at home. Five of these patients had only used the tablet at the hospital, and, of these, only two were able to recall the contents. The same five patients also did not remember being introduced to how to use the tablet
Geerds 2020 [34]	Mobile App for post-op monitoring	No theoretical framework provided	Participants were provided verbal and written instructions for using the app. No further description of the content of the app is provided	Nurses	Web-based	Home	6-months post discharge	Not described	Not described	Of the participants (29/110, 26.4%) who downloaded the mobile app, only 1 (1/29, 3.4%) completed the app questionnaire (used to measure usability of app)
Backman 2020 [36]	MyPath to Home	Use of a user-centered design process, integrated with a modern agile software development methodology	Patients, caregivers, and clinicians received training on how to use the MyPath to Home web-based application prior to obtaining access to it. With the application, patients and their caregivers were able to securely access the discharge records and to access them seamlessly across a number of mobile devices, including smartphones, tablet computers, and laptop computers. The records were synchronized between these devices, helping the patients and their caregiver stay up to date. The five key features included (1) access to a discharge plan upon admission to geriatric rehabilitation; (2) sharing of preferences and needs with the “circle of care” team members; (3) access to multiple resources through the health library (ie, workbooks) on their dashboard; (4) access to their personal rehabilitation goals of care; and (5) access to personalized discharge information including discharge date, follow-up appointments, who to contact, equipment needs, home accommodation, community resources, and list of medications	Patients, caregivers, clinicians (physiotherapists, social workers, occupational therapists, physicians, nurses)	Web-based	Home	30 days post-discharge	Not described	Clinicians can review each of their patient’s specific preferences and needs during their rounds, assign specific resources to the health library (ie, workbooks), and upload all individualized discharge information and resources	Not described
Gao 2021 [29]	WeChat Group	No theoretical framework provided	When control group patients were discharged from the hospital, they were given the usual paper discharge instructions and rehabilitation exercise guidance. In addition to these measures for the observation group, the doctors also added the patients’ WeChat and joined the WeChat group chat formed by the medical team. Doctors transmitted text, pictures, voice and video to the group to guide and urge patients to perform rehabilitation exercises, such as correct sitting posture, when to use abduction, and when to abandon it; patients could also consult their condition and upload their own rehabilitation results through WeChat	Physicians	Web-based	Home	60 days post-discharge	Not specified	For special patients, doctors would provide personalized and targeted guidance, for example, patients who were used to putting the affected limb on the other leg to prevent the prosthesis from coming out were send pictures or videos of correct posture and prohibited actions through WeChat	Not described
Ko 2021 [37]	Rehabilitation instructions after hip fracture surgery	No theoretical framework provided	The contents of the mobile application include rehabilitative exercises, activities of daily living (ADL), pain management, nutrition management, fall prevention, and hospital visits. The selection of rehabilitative exercises and ADL was evidence-based after the first and second authors reviewed the literature and discussed the exercises required after hip fracture surgery. The rehabilitative exercises also include goal setting in which older adults can set a count for bed and standing exercises and a duration for walking exercises for every day. The ADL includes advice on how to use a bed and a toilet, correct posture, and postures to avoid while sitting in a chair, picking up things, taking a shower, cleaning, lying down, wearing pants, washing hair, and sitting in a car. The actions of using a bed and a toilet were shown using a video format, and correct postures and postures to avoid in the form of pictographs for easier understanding. The information on pain and nutrition management, fall prevention, and hospital visits was based on literature reviews and designed as static images and concise, large-sized characters	Clinicians (clinical instructors, orthopedic surgeon, head nurses, orthopedic nurses)	Mobile application	Hospital	Not specified	Not described	Not described	Not described
**Web-based resources (*n* = 2)**										
Nahm 2012a, Nahm 2012b, Nahm 2013 [23–25]	Online hip fracture caregiver resource center (OHRC) program for caregivers	The program was developed in conformance with the stress, appraisal, and coping theory and self-efficacy theory	The online hip fracture CG resource program included seven self-learning modules, moderated discussion boards, an Ask-the-Experts section, and a virtual library	Nurses	Web-based	Hospital and Home	8 weeks	1–2 modules per week	During the orientation session, the project manager (PM), a nurse who had expertise in hip fracture care, and the CG participant developed a weekly course plan based on the specific caregiving needs	Nahm 2012a: A total of 41 dyads were eligible and 36 dyads were enrolled. (See Fig. 1 for an enrollment flow diagram.) Reasons for CR and/or CG refusal to participate in the study during the screening process, which occurred for 37 potentially eligible dyads, are presented in Table 2. The most frequent reasons for refusal for CRs were “not interested” (*n* = 10) and “concerned about CG burden” (*n* = 6), whereas the most frequent reason for CG refusal was “being too busy” (*n* = 12). Other reasons for CG refusal included concerns about additional burden and situations that required the CR’s readmission to the acute care hospital. Nahm 2012b: Among 70 potentially eligible CGs and CRs, three CRs did not respond to follow-up calls for the baseline interviews and 17 CGs and 12 CRs refused to participate in the study. Of the 41 eligible CGYCR dyads, 36 dyads were enrolled (five CRs refused later due to changes in health conditions) and 27 dyads completed the follow-up survey. Most frequent withdrawal reasons were illness of the CR or other family members. Nahm 2013: The majority (n 25; 92.6%) accessed the discussion boards, but only 19 CGs (70.4%) actively posted their thoughts on the discussion topic
Yadav 2021a, Yadav 2021b [26, 27]	Digital patient health hub	Health behavior change supporting systems (HBCSS) in Yadav 2021a No theoretical framework provided in Yadav 2021b	This proposed web-based health information portal, or a website, is intended for patients who can access all relevant information about their hip fractures. It includes details in multimedia formats of diagnosis and treatment options, medications, wound management and rehabilitation exercises, potential problems encountered during the hospital admission and post discharge, information on how to deal with difficulties, as well as how and when to attend follow-up appointments or seek more help from the health care team	Patients, Caregivers, and non-specified clinicians	Web-based	Multiple settings	Not described/applicable	Not described/applicable	It is interactive, enabling patients and their caregivers to provide both targeted and patient-initiated information to their health care clinician	Not described/applicable
**Wearable devices /sensor monitoring (*n* = 1)**										
Pol 2019 [35]	Sensor monitoring system	Cognitive behaviour theory and Bandura’s self-efficacy theory	Three pairs of skilled nursing facilities were randomised to one of three fixed sequences Each sequence started with providing care as usual (the control condition), followed by CBT-based occupational therapy and ending with **CBT-based occupational therapy with sensor monitoring.** Patients in CBT-based occupational therapy with sensor monitoring received the same occupational therapy programme as the first intervention group as well as sensor monitoring The sensor monitoring system consists of a wearable physical activity monitor (PAM AM300) (http://www.coach.com), motion sensors (Molite sensor Z wave Benext, https://www.benext.eu/) placed in the main spaces in the patients’ house and a gateway (Raspberry Pi with a Z-wave shield Model B + quad core CPU, 1024 MB RAM). The PAM measures body movement expressed by the PAM-score and communicates with the gateway via a Bluetooth adaptor (WR300-E). The motion sensors communicate wirelessly through a Z-wave protocol with the gateway. Via a web-application, users can see the visualisations	Occupational Therapists	Face to face and telephone consultations. Monitored activity through web-based app	Rehabilitation and Home	2 ½ months intervention, monitored for 6 months post-discharge	While in the skilled nursing facility, patients received weekly coaching. After discharge, the patients received four home visits followed by four telephone consultations over two and a half months	Not described	Total *n* = 240 at start. During the study, 47, 43 and 22 patients had dropped out after 1, 3 and 6 months, respectively. During admission to the skilled nursing facility, 97.6% patients in the care as usual, 100% patients in the CBT-based occupational therapy and 95.8% patients in the group CBT-based occupational therapy with sensor monitoring received the occupational therapy sessions. The median inpatient number of sessions was 4 (IQR 2–5) for the care as usual, 4 (IQR 2–6) for the CBT-based occupational therapy and 2.5 (IQR 1–5) for the CBT-based occupational therapy with sensor monitoring. At home, the median number of occupational therapy sessions (range 1–4) was 2 (IQR 0–4) for CBT-based occupational therapy and 4 (IQR 2–4) for CBT-based occupational therapy with sensor monitoring. The median duration of sessions at home was 41 (IQR 0–60) minutes for CBTbased occupational therapy and 45 (IQR 38.5–60) minutes for CBT-based occupational therapy with sensor monitoring

### Outcome measures

The quantitative outcomes reported consisted of patient-related functional outcomes (*n* = 6), gait/mobility (*n* = 7), quality of life (*n* = 2), psychological factors (*n* = 3), and survival/complications (*n* = 1). Other quantitative reported outcomes included compliance (*n* = 2), technology-user interactions (*n* = 4), and caregiver outcomes (*n* = 3). The qualitative results from 6 studies [18, 26, 27, 33, 36, 37] included feedback from participants on the use of the digital health interventions. Overall, the feedback provided areas for improvement as well as benefits to the use of these interventions. Specifically, three studies included feedback from clinicians [27, 36, 37], two explored specifically the needs of caregivers [19, 25] and one study described the challenges with the study recruitment processes [23]. All the study outcomes are found in Table 3, 4 and 5.

**Table 3:** Patient-related study outcomes (quantitative)

Lead author (year)	Name of Digital Health Intervention	Functional Outcomes	Gait / Mobility	Quality of Life	Psychological factors	Survival / Complications	Direction and magnitude of effect
Ortiz-Pina 2021 [20]	@ctivehip	S	Mixed	-	-	-	**Functional Outcomes** **Function Independent Measure (FIM) + ,** high effect size: 1.06 Cohen’s d; *p* < 0.001 (S) **Gail/Mobility** **Time-Up and Go (TUG)** + , high effect size: 0.95 Cohen’s d; *p* = 0.001 (S) **Short Physical Performance Battery (SPPB)** + , 0.48 Cohen’s d; *p* = 0.067 (NS)
Mora-Traverso 2022 [21]	@ctivehip	-	S	S	S	-	**Gail/Mobility** **Fitness level** + , medium effect size: 0.70 Cohen’s d; *p* = 0.008 (S) **Quality of Life** **EuroQol Quality of Life Questionnaire (EQ-5D) total perceived health index** + , medium effect size: 0.67 Cohen’s d; *p *= 0.010 (S) **Psychological factors** **Hospital Anxiety and Depression Scale (HADS) total score** + , medium effect size: 0.70 Cohen’s d; *p* = 0.007 (S)
Bedra 2015 [22]	Hip Hat System	Mixed	Mixed	Mixed	Mixed	-	**Functional Outcomes** **Modified Barthel Index** + , t = 1.87, *p* = 0.10 (NS) **Lower Extremity Functional Scale (LEFS)**. + , t = 2.58, *p* = 0.03 (S) **Gail/Mobility** **Energy Expenditure from Yale Physical Activity Survey (YPAS) (kcal/d) subscale** + , t = 0.7, *p* = 0.5 (NS) **Total Time (hours/wk) subscale** + , t = 2.49 (effect), *p* = 0.04 (S) **Quality of Life** **SF-36 Physical Functioning subscale** pre-test (38 ± 27), post-test (71 ± 31): t = 3.48 (effect), p = 0.009 (S) **Role limitations due to physical health problems subscale** pre-test (6 ± 10), post-test (17 ± 12): t = 2.03 (effect), *p* = 0.05 (S) **Role limitations due to emotional problems subscale** pre-test (22 ± 6), post-test (23 ± 6): t = 0.43 (no effect), *p* = 0.68 (NS) **Vitality subscale** pre-test (64 ± 20), post-test (74 ± 25): t = 1.58 (no effect), *p* = 0.15 (NS) **Mental Health subscale** pre-test (83 ± 15), post-test (88 ± 12): t = 0.93 (no effect), *p* = 0.38 (NS) **Social Functioning subscale** pre-test (54 ± 31), post-test (85 ± 28): t = 3.27 (effect), *p* = 0.01 (S) **General health subscale** pre-test (78 ± 18), post-test (86 ± 18): t = 1.60 (no effect), *p* = 0.15 (NS) **Health Transition subscale** pre-test (47 ± 40), post-test (22 ± 18): t = -2.12 (effect), *p* = 0.05 (S) **Psychological factors** **Center for Epidemiological Studies Depression Scale** pre-test (9 ± 10), post-test (8 ± 9): t = -0.80 (no effect), *p* = 0.45 (NS) **Mini Mental Status Examination (MMSE)** pre-test (27 ± 2), post-test (28 ± 2): t = 1.12 (no effect), *p* = 0.29 (NS) **Exercise Self-Efficacy scale** pre-test (6 ± 3), post-test (9 ± 1): t = 3.16 (effect), *p* = 0.01 (S)
Morris 2021 [28]	Telerehabilitation (TR)	Clinically important difference (MCID)	Clinically important difference (MCID)	-	-	-	**Functional Outcomes** **Total FIM** at admission (95.71 ± 14.03), discharge (105.94 ± 12.77) **Gail/Mobility** **Timed Up and Go (TUG)** + , 43% change (− 16.62 ± 18.13 s) which is greater than the MCID of 31% **De Morton’s Mobility Index (DEMMI)** + , 10 point (10.12 ± 8.66), which is greater than the MCID of 6 points
Nahm 2012b [24]	Online hip fracture resource center (OHRC)	-	S	-	NS	-	**Gail/Mobility** **Physical activity (kcal)** -, t = 2,73, *p* = .01 from the month prior to hip fracture to the 8-week follow-up period (S) **Psychological factors** **Self-Efficacy for Exercise Scale** + , t = 1.49, *p* = 0.15 (NS)
Pol 2019 [35]	Sensor monitoring informed occupational therapy on top of cognitive-behavioral treatment (CBT) based coaching program	S	-	-	-	-	**Functional outcomes** **Daily functioning (measured using the Canadian Occupational Performance Measure COPM)** 1) **performance** + , difference 1.17 [95% CI (0.47–1.87) *P* = 0.001 (S) 2) **satisfaction** + , difference 0.94 [95% CI [0.37–1.52] *P* = 0.001 (S)
Gao 2021 [29]	WeChat Group	NS	-	-	-	S	**Functional outcomes** **Harris hip Score (HHS)** + , t = 4.776, *p* = 0.000 (NS) **Survival/Complications** **Mortality** -, (*n* = 1 (observation group) vs *n* = 6 (control group), *p* = 0.048 (S) **Complications** -, (*n* = 6 (observation group) vs *n* = 15 (control group) at 60 days post-discharge, *p* = 0.022 (S)
Cheng 2022 [31]	Mobile App	-	NS	-	-	-	**Gail/Mobility** **Modified functional ambulatory category (MFAC)** (*p* = 0.728) (NS) **Elderly Mobility scale (EMS)** (*p* = 0.647) (NS) **Lower extremity functional scale (LEFS)** (*p* = 0.411) (NS)
Li 2022 [30]	CASPAR Health e-system	NS	NS	-	-	-	**Functional Outcomes** **Modified Barthel Index (MBI)** (*p* > 0.05) (NS) **Lawton Instrumental Activities of Daily Life scale** (*p* = 0.626) (NS) **post test Lawton IADL scale:** experimental group = + 4, control group = + 1.15 (NS) **follow-up Lawton IADL scale:** experimental group = + 2.9, control group = + 0.95 (NS) **Gail/Mobility** **Time Up and Go (TUG), Functional Reach test (FR), Pain Visual Analogue Scale (VAS), and Fall Efficacy Scale (FES)** (*p* > 0.05) (NS) **Morse Fall Scale (MFS)** (*p* = 0.563) (NS)
Kalron 2018 [38]	Telerehabilitation program based on a video platform for therapy software program	-	+	-	-	-	**Gail/Mobility** Greater improvements in the telerehabilitation group were demonstrated in the 2-min walking test (86.1%) and walking speed (65.6%)

**Table 4:** Other study outcomes (quantitative)

Lead author (year)	Name of Digital Health Intervention	Compliance	Technology-user interactions	Caregiver related outcomes	Direction and magnitude of effect
Bedra 2015 [22]	Hip Hat System	-	S	-	**Technology-user interactions** **Client Satisfaction Questionnaire-8 (CSQ-8)** + , pre-test (27 ± 4), post-test (31 ± 0.46) t = 2.47, *p* = 0.04 (S)
Nahm 2012b [24]	Online hip fracture resource center (OHRC)	NS	+	Mixed	**Compliance** **Self-Efficacy for Osteoporosis Medication Adherence (SEOMA)** (t = 1.54, *p* = 0.14) (NS) **Technology-user interactions** **Perceived Health Web Site Usability Questionnaire (PHWUQ)** 74.04 ± 7.26 (range, 58–84) **Caregiver related outcomes** **eHealth literacy Scale** + , t = 2.43, *p* = .022 (S) **Computer-mediated functional social support scale** + , t = 0.26, *p* = 0.800 (NS) **Rhode Island Stress and Coping Inventory** + , t = 1.63, *p* = .116 (NS) **Computer-mediated social network scale** + , t = 0.61, *p* = .547 (NS) **Knowledge about caring for hip fracture patients on the learning modules questionnaire** + , t = 3.17, *p* = .004 (S)
Gao 2021 [29]	WeChat Group	-	S	-	**Satisfaction** + , t = 314, *p* = 0.007 (S)
Cheng 2022 [31]	Mobile App	NS	**-**	S	**Compliance** **Exercise adherence (second month)** + , *p* = 0.09 (NS) **Caregiver related outcomes** **Modified caregiver strain index (M-CSI)** -, *p* = 0.531 (NS)
Backman 2020 [36]	MyPath to Home	-	+	-	**Technology-user interactions** **Technology readiness index (TRI) 2.0** 3.26 / 5, moderate level of technological adoption **Satisfaction** easy to understand (21/23, 91%), helpful (21/23, 91%), helped to understand what they needed to do to prepare for discharge (22/23, 96%), helped to identify the skills they needed to have for a successful discharge (20/23, 87%) 78% (18/23) found that the organization of the application made sense and that it was easy to navigate 91% (21/23) would recommend this application to other patients
Kalron 2018 [38]	Telerehabilitation program based on a video platform for therapy software program	+	-	-	**Compliance** 66.7% in telerehabilitation group (10/15) performed at least 15 of 18 exercise sessions compared with only 41.0% (7 of 17)

**Table 5:** Study outcomes (qualitative) (*n* = 9)

Lead author (year)	Name of Digital Health Intervention Participants (n)	Participants	Categories	Themes
Ariza-Vega 2021a [18]	@ctivehip	Caregivers	Feedback on the usefulness of the program	(1) the telerehab program was perceived to be useful for older adults’ functional recovery without being onerous for family (2) there was room for improvement in the telerehab program (regular checking and monitoring by health professionals, more variety of exercises, difficulty of exercises, limited internet access in some locations, no (3) positive points to program (good for communication with health professionals, easy to use, helpful)
Ariza-Vega 2021b [19]	@ctivehip	Caregivers	Needs of caregivers (Perceptions regarding the hip fracture and recovery process and reasons for choosing or declining the telerehab program)	Caregivers’ responses to the hip fracture and recovery process: (1) concern about survival and recovery (2) uncertainty, anxiety, and stress (3) communication and resources: looking for answers The reasons for choosing the telerehab program were: (1) to enhance recovery after fracture, (2) gain knowledge for managing at home, and (3) the convenience of doing exercises at home The reasons for declining the telerehab program were: (1) perceived challenges with technology; (2) lack of time to support family member (with hip fracture) with technology, for example, navigating the website; (3) caregivers’ perception that family members would not want to complete exercises at home; (4) preference of in-person rehab, even if it had associated costs; or (5) no expected need for the program
Nahm 2012a [23]	Online hip fracture caregiver resource center (OHRC)	Patients and caregivers	Challenges and strategies to recruiting older adult hip fracture patients, and caregivers	Challenges: Identifying eligible dyads: we found that locating family CGs often required additional assistance from the clinical staff since CGs were often not present when the research nurse visited the patient on the unit and the clinical; Composite Eligibility Criteria for the Dyad: During the early recruitment phase, we found that the number of eligible dyads in the selected three inner-city hospitals was smaller than expected; Brief Hospital length of stay: Enrollment of older adult hip fracture patients and their CGs in this acute phase is challenging and resource intensive. Usually, our research nurse did not have an opportunity to introduce the study to the patient until the second visit; Caregivers’ Stress Level and Busy Schedule: These CGs were stressed and physically and mentally exhausted. Thus, some CGs perceived participation in an online study to be an additional stressor and burden Strategies: Identifying eligible dyads: Informing necessary clinical staff about the study and establishing rapport between the study field staff and the clinical staff are critical in recruiting dyads; Composite Eligibility Criteria for the Dyad: Our investigative team confirmed the importance of monitoring and developing strategies for the recruitment process; Brief Hospital length of stay: Support from the hospital staff was especially helpful in dealing with the patient’s short hospital stay and the dyads’ busy schedules; Caregivers’ Stress Level and Busy Schedule: Field research nurses must be appropriately trained to understand the situation and make proper judgments when they approach participants
Nahm 2013 [25]	Online hip fracture caregiver resource center (OHRC)	Caregivers	Needs of caregivers (Description of caregiver activities, strategies and coping mechanisms used by caregivers)	(1) Description of caregiving activities help with ADLs or physical therapies; environmental adjustment; provision of direct care related to the surgery (e.g., medications, care of localized infection site); and use of assistive devices (2) Strategies Used by Caregivers to Prevent Hip Fractures The most frequently discussed strategies related to safety, such as becoming aware of surroundings and being careful not to fall (nine units) “I am trying very hard to keep my hubby from falling again. I know that I am being very overprotective, but he just can’t afford another fall.” The impact of knowledge gained by caregivers also expanded to themselves, as well as to their family members (3) Coping Mechanisms Used by the Caregivers to Handle Stress Several caregivers (four units) reported that support from their family and friends helped them a great deal to cope with the stressful situation (“I’ve found that lots of family support and visits from friends helped both of us to cope”). Others found that relaxation techniques, exercise or taking a walk, or reading helped them cope with the stress (five units)
Yadav 2021a [26]	Digital patient health hub	Caregivers	Feedback on the application	(1) Context patient characteristics such as frailty, digital literacy, and patient or carer participation, social support, whereas healthcare delivery aspects included the structure and culture of existing practice and the need for innovation and holistic models of care (2) Content importance of targeted patient education and behavior change (3) System personalization across modes of content delivery. This must foster trust, ensure adequate financing, and support ownership and privacy by establishing appropriate mechanisms for embedding change
Yadav 2021b [27]	Digital patient health hub	Patients, caregivers and staff	Feedback on the application (Barriers and facilitators influencing the use of the application)	(1) Capability: Some patients recognized that possessing the necessary knowledge and skills while accessing digital devices can help explore relevant web-based health information, which could enable a better understanding of their health condition. Conversely, there were caregivers who lacked confidence in using digital devices (2) Opportunity: Patients and their family members considered their personal environment and the affordability of resources, such as digital devices, as a major limiting factor. Caregivers saw digital health platforms as an opportunity to provide general health information, including healthy lifestyle, diet, and exercise Motivation: Being older was identified by both patients and their family members as one of the main hurdles. (3) Caregivers identified lack of time in their existing role, which is currently not a part of their job. Participants across all 3 categories identified their existing capabilities as a limiting factor. However, they were also positive about the potential capabilities of a digital solution, such as the availability of information that would reduce the need to visit a physician and access to trustworthy interventions. Staff thought that a digital health solution could potentially improve handover processes through a better exchange of information between specialists and caregivers. Most participants were optimistic about the range of functions that a digital health platform could provide; however, some had reservations such as preferring phone conversations or maintaining conventional face-to-face interactions with the physician. Emotionally, some consumers were unhappy with the services provided through technology-based solutions in comparison with face-to-face interactions. One of the patients identified a potential lack of reinforcement in terms of someone who could teach or handhold, which could be a barrier to using a digital solution. Conversely, some patients thought that it could help them achieve more peace of mind and service satisfaction
Jensen 2019 [33]	My Hip Fracture Journey	Patients and caregivers	Feedback on the application (Perspectives of participants on feeling supported or not by the technological solution)	(1) regaining physical ability, (2) support of autonomy, (3) the issue of getting old, (4) usability of the tablet and app, and (4) uncertainty about the future and general attitude toward life
Backman 2020 [36]	MyPath to Home	Patients, caregivers, and clinicians	Feedback on the application (Challenges and benefits to the use of the application)	Participants described that an application, like MyPath to Home, was essential to help manage the personalized needs of geriatric rehabilitation patients during their transition from the hospital to home (1) Challenges included the application adding to their workload, a need for more education about the application, and a need for the app to be more user-friendly and accessible on more devices (2) Benefits included providing the patient with opportunities to be involved in their care
Ko 2021 [37]	Rehabilitation instructions after hip fracture surgery	Clinicians	Feedback on the application	1) The application is concise and simple, 2) The video of rehabilitation exercises is easy and helpful for older adults to follow, 3) A function to replay the rehabilitation exercise video is needed, 4) It is necessary to reorganize the exercises by dividing bed exercises into lying and sitting exercises, 5) Goal setting can be difficult for older adults. 6) It is necessary to maximize the font size for goal setting, 7) Some of the video quality needs to be improved, 8) The background color of the application is suitable, but the yellow text is not easy to read

### Barriers and enablers

Sixteen studies identified key barriers and enablers to the use of digital health interventions [18–20, 23–31, 33, 34, 36, 37]. For clinicians, a key barrier to the use of the digital health interventions was the *acceptability of the technology* [27, 36]. However, the *usefulness of the digital health intervention* by clinicians was seen as both a barrier and an enabler [26, 27, 29, 37]. For patients and caregivers, all the themes were seen as both a barrier and an enabler depending on the study. Some patients and caregivers saw the *lack of availability and access to the digital health intervention* as a barrier [18, 26, 27, 30, 31, 33, 36] but others had no problems with the *availability or access to the intervention* [18, 26]. Patients and caregivers described *limited usability of the technology* [19, 30, 34] where others had *no concerns with the usability* [18, 24, 28, 36, 37]. There were also conflicting views with the patients and caregivers perceived *knowledge and skills to the use the technology*, as a barrier [18, 19, 23, 24, 26–28, 30, 31, 33, 34] or an enabler [18, 20, 28]. Patients and caregivers described mixed views on the *acceptability of the technology* as a barrier [19, 26–28] or enabler [19, 25–27, 33] and the *usefulness of the information* as a barrier [18, 19, 26, 31, 37] or enabler [18, 19, 24, 25, 36]. Details of the barriers and enablers as well as the proposed behavioural change techniques are described in Table 6.

**Table 6:** Barriers and enablers to the use of patient-clinician digital health interventions for older patients with a hip fracture transitioning from hospital to rehabilitation to home

Themes	TDF Domains	Barriers	Enablers	BCTs (Behaviour Change Techniques)
**Patients and Caregivers**				
**Availability and access to the digital health intervention**				
No access to technology or internet in some locations	Environmental context and resources	Ariza-Vega 2021a [18], Yadav 2021a [26], Jensen 2019 [33], Backman 2020 [36]		Restructuring the physical environment
Devices need to be large enough to view content	Environmental context and resources	Li 2022 [30], Cheng 2022 [31]		Restructuring the physical environment
No problems related to internet access	Environmental context and resources		Ariza-Vega 2021a [18], Yadav 2021a [26]	Restructuring the physical environment
Affordability of device needs to be considered	Environmental context and resources	Yadav 2021b [27]		Restructuring the physical environment
**Usability of the digital health intervention**				
Some reports of technology problems, perceived challenges with technology, participants reported challenges in adoption of the technology	Environmental context and resources and skills	Ariza-Vega 2021b [19], Geerds 2020 [34], Li 2022 [30]		Restructuring the physical environment
Having the intention to use the application	Intentions	Geerds 2020 [34]		Commitment
Low download rate of mobile app and potential need for more education	Knowledge and skills	Geerds 2020 [34]		Practice and feedback
Digital intervention (app) is easy to understand and navigate, user-friendly	Reinforcement		Backman 2020 [36], Ko 2021 [37], Ariza-Vega 2021a [18], Nahm 2012b [24], Morris 2021 [28]	Incentive
Digital intervention (app) has consistency in design	Reinforcement		Ko 2021 [37]	Incentive
Belief that other patients could easily use app	Beliefs about consequences		Morris 2021 [28]	Social and environmental consequences
**Knowledge and skills to use the digital health intervention**				
Potential challenges with adoption of technology	Environmental context and resources	Ariza-Vega 2021b [19], Geerds 2020 [34]		Restructuring the physical environment
Lacking confidence in abilities to use technology	Beliefs about capabilities	Yadav 2021b [27]		Verbal persuasion to boost self-efficacy
Comfortable feeling with technology/app	Beliefs about capabilities		Morris 2021 [28]	Verbal persuasion to boost self-efficacy
Belief that being of older age limits technology use	Belief about capabilities, Professional role and identity	Yadav 2021a [26], Yadav 2021b [27], Jensen 2019 [33]		Verbal persuasion to boost self-efficacy
Online interventions seen as burdensome	Optimism	Nahm 2012a [24]		Verbal persuasion to boost self-efficacy
Knowledge and skills needed to use technology	Knowledge and skills	Yadav 2021b [27], Li 2022 [30]		Practice and feedback
Not having caregivers to support use of technology is an issue	Environmental context and resource, social influence	Yadav 2021b [27]		Restructuring the social environment
Not seen as onerous/time consuming for caregivers, importance of support of caregivers	Beliefs about consequences		Ariza-Vega 2021a [18], Ortiz-Pina 2021 [20]	Social and environmental consequences
Patients required high levels of help to use digital intervention	Environmental context and resources	Geerds 2020 [34], Cheng 2022 [31]		Prompts/cues
Additional responsibilities for caregivers, lack of time to support family member, caregivers stress level and busy schedule, participation as an extra burden during an already difficult time, residential caregivers note time constraints	Beliefs about consequences	Ariza-Vega 2021a [18], Ariza-Vega 2021b [19], Nahm 2012a [23], Yadav 2021b [27]		Social and environmental consequences
Digital literacy and knowledge of technology is variable and/or lacking	Knowledge and skills	Yadav 2021a [26], Jensen 2019 [33]		Practice and feedback
Lack of interest in technology use	Beliefs about consequences	Jensen, 2019 [33], Morris 2021 [28]		Social and environmental consequences
Unable to use app and/or remember information due to stress, fatigue, cognition or too much information received at once, feeling overwhelmed	Beliefs about capabilities, Memory	Geerds 2020 [34], Yadav 2021a [26], Jensen 2019 [33], Morris 2021 [28]		
**Acceptability of the digital health intervention**				
Caregivers saw platform as being an easier option to provide resources and videos	Beliefs about consequences		Yadav 2021b [27]	Pros and cons
Preference for face-to face for all patients, preference of in-person rehab, even if it had associated costs, no expected need for the program, desiring traditional rehab instead	Beliefs about consequences	Ariza-Vega 2021b [19], Morris 2021 [28], Yadav 2021b [27]		Social and environmental consequences
Positive feelings about the potential capabilities and utilities of a digital solution	Beliefs about consequences, Emotions		Yadav 2021b [27], Nahm 2013 [25]	Social and environmental consequences
Need for change in mindset of technology advancements	Beliefs about consequences	Yadav 2021a [26]		Social and environmental consequences
Potential in availability of information to limit going out for physician appointments or convenience of exercise training at home	Beliefs about consequences		Yadav 2021b [27], Ariza-Vega 2021b [19]	Pros and cons
Better engagement in conversations with their clinicians about their care processes	Beliefs about consequences		Yadav 2021a [26]	Pros and cons
Positive about receiving information from peers (e.g. discussion boards) rather than only clinicians	Emotions		Jensen 2019 [33]	Social support
Concerns with ownership and data security	Beliefs about consequences	Yadav 2021a [26]		Social and environmental consequences
**Usefulness of the digital health intervention**				
Participants were pleased with program content (including exercises)	Reinforcement		Ariza-Vega 2021a [18]	Incentive
Participants requested more variety in program contents (exercise) needed	Reinforcement	Ariza-Vega 2021a [18]		Incentive
Participants felt information is easy to understand	Reinforcement		Backman 2020 [36]	Incentive
Participants felt app was comprehensive and helpful for others	Reinforcement		Nahm 2012b [24]	Incentive
Participants stated a need for more community resources on the app	Reinforcement	Yadav 2021a [26]		Incentive
Participants desired content needs to be more tailored to individual needs	Reinforcement	Yadav 2021a [26]		Incentive
Participants requested larger font size and appropriate colour needed	Reinforcement	Li 2022 [30], Ko 2021 [37], Cheng 2022 [31]		Incentive
Gained knowledge for managing at home	Reinforcement		Ariza-Vega 2021b [19]	Incentive
Knowledge and eHealth literacy improved with intervention for caregivers and expanded to their family members	Knowledge and skills		Nahm 2012b [24], Nahm 2013 [25]	Practice and feedback
Perception that patient would not want to complete exercises	Optimism	Ariza-Vega 2021b [19]		Verbal persuasion to boost self-efficacy
Helped understand what they needed to prepare for discharge, helped identify skills they needed for successful discharge	Knowledge and skills		Backman 2020 [36]	Practice and feedback
**Clinicians**				
**Acceptability of the digital health intervention**				
Lack of confidence in using web-based app	Beliefs about capabilities	Yadav 2021b [27]		Verbal persuasion to boost self-efficacy
More education to use digital intervention is needed	Knowledge and skills	Backman 2020 [36]		Practice and feedback
Time consuming to use	Beliefs about consequences	Backman 2020 [36]		Practice and feedback
**Usefulness of the digital health intervention**				
Could improve communication between clinicians and caregivers	Beliefs about consequences		Yadav 2021a [26]	Pros and cons
Could be helpful for new clinicians	Beliefs about consequences		Yadav 2021b [27]	Pros and cons
Application is concise and simple	Environmental context and resources		Ko 2021 [37]	Prompts/cues
Rehabilitation video exercises are easy and helpful for older adults to follow	Environmental context and resources, Beliefs about consequences		Ko 2021 [37]	Prompts/cues
Font colour is difficult to read	Environmental context and resources	Ko 2021 [37]		Restructuring the physical environment
Accuracy of information is important	Environmental context and resources	Ko 2021 [37]		Restructuring the physical environment
Chat feature allowed clinicians to guide and urge patients to exercise, and answer their doubts, and reduce the pressure of patients with medical difficulties	Reinforcement		Gao 2021 [29]	Incentive

## Discussion

A total of 21 studies were included in this scoping review. Of the 21 studies, we identified 14 distinct patient-clinician digital health interventions for post-surgery hip fracture patients including telehealth /telerehabilitation programs (*n* = 6), care transition /follow-up interventions (*n* = 5), online resources (*n* = 2), and wearable devices /sensor monitoring (*n* = 1). Many interventions focused only on a few post-hip fracture surgery care components such as rehabilitation exercises (*n* = 13), follow-up and management (*n* = 9), post-hip fracture education and self-care (*n* = 4), and caregiver needs (*n* = 3) rather than on more comprehensive post-care efforts. In addition, we found that the interventions all existed within a specified team within a particular organization rather than across different organizations, similarly to what others found [40]. Furthermore, these interventions often (or all) lacked complete descriptions following the TidieR guidelines [14] to allow clinicians to use or to allow researchers to replicate the studies. Overall, more clear descriptions of the interventions are needed so that they can be replicated.

In terms of the functions of the digital health tools used in the included studies, the interventions focused primarily on management and informational categories of the continuity of care framework [39]. However, future studies should consider incorporating the relational continuity as an important component of continuity of care.

Although only 3/21 studies in our review specifically included methods to improve clinician engagement, one recent rapid review of transition-focused digital health interventions specifically highlighted the importance of involving clinicians in the design and implementation of these interventions to ensure better uptake [41]. Similarly, another review recommended to better engage clinicians in the design and implementation of technologies [42]. Further considerations should also be made to inform patients on how to use digital health technology, provide appropriate training to clinicians, and ensure that the adoption of the technology will allow clinicians more time to care for patients [42].

In our scoping review, we were also able to identify key barriers and enablers to the uptake of digital health interventions. The unique key barrier was the acceptability of the technology by the clinicians. Thus, the behavioural change techniques [17] related to this barrier are *practicing and giving feedback* and *utilizing verbal persuasion to boost self-efficacy*. The behavioural change techniques [17] were also matched to the other barriers and enablers and consist of *restructuring the physical and social environments*, *providing incentives*, *identifying social and environmental consequences*, *explaining pros and cons*, *providing prompts/cues*, and *providing appropriate social support*. Future studies should consider the inclusion of these behavioural change techniques in the implementation strategies of digital health interventions.

### Strengths and limitations

The broad inclusion criteria for this scoping review allowed us to examine a wide variety of patient-clinician digital health interventions for the hip fracture population. However, the studies lacked detailed description of the interventions. However, despite the wide-ranging inclusion criteria, it is possible that some studies with non-significant results were not published.

## Conclusion

In our scoping review, we identified existing patient-clinician digital health interventions. The findings highlighted many behavioural factors that could affect the uptake and use of these patient-clinician digital health interventions. However, a specific attention should be focused on the acceptability of the technology by the clinicians to encourage uptake of the digital health interventions. The results of this scoping review can help researchers and clinicians to better understand the key factors that can be targeted to help increase the uptake of technology-based intervention use by clinicians, patients, and caregivers. Further research is needed to look at patient-clinician digital health interventions in different patient populations that span across different health care sectors.

### Supplementary Information

**Additional file 1: Appendix 1.** Search strategies.

**Additional file 2: Appendix 2.** Excluded studies (*n*=25).

## Data Availability

All data generated or analysed during the current study are included in this published article and its supplementary files.
