# Supplementary material for: Patient-clinician digital health interventions for the hip fracture population: a scoping review
Source: BMC Health Serv Res. 2023 Oct 2;23:1052. doi: 10.1186/s12913-023-09784-y (PMC10546736; doi:10.1186/s12913-023-09784-y)
Supplement: Supplementary file 1 — Additional file 1: Appendix 1. Search strategies. [file 12913_2023_9784_MOESM1_ESM.docx]

**Appendix 1_Search Strategies**

2022 May 15

Ovid Multifile

Database: Embase Classic+Embase <1947 to 2022 May 13>, Ovid MEDLINE(R) ALL <1946 to May 13, 2022>, APA PsycInfo <1806 to May Week 2 2022> , EBM Reviews - Database of Abstracts of Reviews of Effects <1st Quarter 2016>, EBM Reviews - Cochrane Central Register of Controlled Trials <April 2022>, EBM Reviews - Cochrane Database of Systematic Reviews <2005 to May 11, 2022>

Search Strategy:

--------------------------------------------------------------------------------

1 exp Hip Fractures/ (74849)

2 ((hip or hips or femoral neck or (femur? adj2 neck?) or acetabul* or intertrochanter* or inter-trochanter* or pertrochanter* or per-trochanter* or subtrochanter* or sub-trochanter* or trochanter*) adj3 (break* or broke* or fractur*)).tw,kw,kf. (93947)

3 or/1-2 [HIP FRACTURES] (111241)

4 exp Telemedicine/ (114712)

5 (telemed* or tele-med* or telecare or tele-care or teleconsult* or tele-consult* or teleconferenc* or tele-conferenc* or telecounsel* or tele-counsel* or telehealth* or tele-health* or telemonitor* or tele-monitor* or telepsychiatr* or tele-psychiatr* or telepsycholog* or tele-psycholog* or telerehab* or tele-rehab* or telesupport* or tele-support* or teletherap* or tele-therap* or teletreatment* or tele-treatment*).tw,kw,kf. (98347)

6 (ehealth* or e-health* or mhealth* or m-health* or emental health* or e-mental health* or mmental health* or m-mental health* or epsychiatr* or e-psychiatr* or mpsychiatr* or m-psychiatr* or epsychol* or e-psychol* or mpsychol* or m-psychol* or erehab* or e-rehab* or mrehab* or m-rehab* or etherap* or e-therap* or esupport* or e-support* or msupport* or m-support* or evisit* or e-visit* or mvisit* or m-visit*).tw,kw,kf. (44970)

7 (ecoach* or e-coach*).tw,kw,kf. (368)

8 (emedicine* or e-medicine*).tw,kw,kf. (296)

9 (mobile health* or mobile care or mobile counsel* or mobile medicine or mobile psychiatr* or mobile psycholog*).tw,kw,kf. (17596)

10 ((digital* or virtual* or remote*) adj3 (care or health* or healthcare or health-care)).tw,kw,kf. (34640)

11 ((digital* or virtual* or remote*) adj3 (appointment* or clinic or clinics or coach* or communicat* or conferenc* or consult* or followup or follow-up or hub or hubs or interven* or manag* or meet* or monitor* or rehab* or support* or therap* or tool or tools or treatment? or visit*)).tw,kw,kf. (96038)

12 (e-provider? or e-clinician? or e-counsel?or? or e-doctor? or e-nurse? or e-physician? or e-practitioner? or e-therapist? or m-provider? or m-clinician? or m-counsel?or? or m-doctor? or m-nurse? or m-physician? or m-practitioner? or m-therapist?).tw,kw,kf. (373)

13 (mobile provider? or mobile clinician? or mobile counsel?or? or mobile doctor? or mobile nurse? or mobile physician? or mobile practitioner? or mobile therapist?).tw,kw,kf. (176)

14 Internet-Based Intervention/ (2113)

15 exp Therapy, Computer-Assisted/ (61754)

16 ((internet* or app or apps or computer* or cyber* or e-application? or e-mail* or email* or electronic mail* or iphone? or i-phone? or (mobile adj2 application?) or mobile-based or mobile phone? or online or (patient? adj3 portal*) or instant* messag* or (secure* adj3 communicat*) or (secure* adj3 messag*) or (secure* adj3 platform*) or (secure* adj3 portal*) or service or services or smarthome* or smart-home* or smarthub? or smart-hub? or smartphone* or smart phone* or technolog* or telecommunicat* or tele-communicat* or telephon* or textmessag* or text-messag* or video* or web or webbased or web-based or webdeliver* or web-deliver* or "web 2.0") adj3 (care or health care or health-care or healthcare or appointment* or coach* or conferenc* or consult* or interven* or manag* or meet* or monitor* or rehab* or support* or therap* or treatment? or visit*)).tw,kw,kf. (707884)

17 (internet* or app or apps or computer* or cyber* or e-application? or e-mail* or email* or electronic mail* or iphone? or i-phone? or (mobile adj2 application?) or mobile-based or mobile phone? or online or (patient? adj3 portal*) or instant* messag* or (secure* adj3 communicat*) or (secure* adj3 messag*) or (secure* adj3 platform*) or (secure* adj3 portal*) or smarthome* or smart-home* or smarthub? or smart hub? or smartphone* or smart phone* or technolog* or telecommunicat* or tele-communicat* or telephon* or textmessag* or text-messag* or video* or web or webbased or web-based or webdeliver* or web-deliver* or "web 2.0").ti,kw,kf. (1010965)

18 (internet* or app or apps or computer* or cyber* or e-application? or e-mail* or email* or electronic mail* or iphone? or i-phone? or (mobile adj2 application?) or mobile-based or mobile phone? or online or (patient? adj3 portal*) or instant* messag* or (secure* adj3 communicat*) or (secure* adj3 messag*) or (secure* adj3 platform*) or (secure* adj3 portal*) or smarthome* or smart-home* or smarthub? or smart hub? or smartphone* or smart phone* or technolog* or telecommunicat* or tele-communicat* or telephon* or textmessag* or text-messag* or video* or web or webbased or web-based or webdeliver* or web-deliver* or "web 2.0").ab. /freq=2 (1141672)

19 or/4-18 [DIGITAL CARE, PT 1] (2342316)

20 exp Hip Fractures/pc, rh, su, th [Prevention, Rehabilitation, Surgery, Therapy] (32838)

21 Delivery of Health Care/ (269689)

22 exp *Delivery of Health Care/ (1485247)

23 Delivery of Health Care, Integrated/ (25449)

24 ((deliver* or model or models or provid* or provision) adj3 (care or health care or health-care or healthcare)).ti,kw,kf. (100050)

25 Health Services/ (197647)

26 Community Health Services/ (89281)

27 Rural Health Services/ (27382)

28 Suburban Health Services/ (177602)

29 Urban Health Services/ (161267)

30 (healthservice* or health service* or healthcare service? or health care service?).ti,kw,kf. (143922)

31 Geriatric Nursing/ (27030)

32 Home Nursing/ (75362)

33 ((geriatric* or home) adj3 (care or health care or health-care or healthcare)).tw,kw,kf. (114734)

34 ((after operati* or after surger* or postoperati* or post-operati* or postsurg* or post-surg*) adj3 (care or health care or health-care or healthcare or followup or follow-up or rehab* or therap* or treatment*)).tw,kw,kf. (212983)

35 Rehabilitation/ (147097)

36 Rehabilitation Nursing/ (3056)

37 exp Exercise Therapy/ (170873)

38 ((exercis* or remedial* or rehab*) adj3 therap*).tw,kw,kf. (70823)

39 rh.fs. [Rehabilitation - Floating Subheading] (394686)

40 rehab*.ti,kw,kf. (282667)

41 rehab*.ab. /freq=2 (194601)

42 Caregivers/ (158043)

43 Family/ (242244)

44 Ambulatory Care/ (98887)

45 (ambulatory adj3 (care or health care or health-care or healthcare)).tw,kw,kf. (40052)

46 Inpatients/ (209614)

47 inpatient*.tw,kw,kf. (419073)

48 Outpatients/ (160456)

49 (outpatient* or out patient*).tw,kw,kf. (689770)

50 Communication/ (270458)

51 communicat*.ti,kw,kf. (269927)

52 communicat*.ab. /freq=2 (271887)

53 (miscommunicat* or mis-communicat*).tw,kw,kf. (3545)

54 (misunderstand* or mis-understand*).tw,kw,kf. (22723)

55 (misinform* or mis-inform*).tw,kw,kf. (13853)

56 Information Seeking Behavior/ (7412)

57 ((communicat* or provid* or provision* or seek* or search* or shar* or sought) adj3 information).tw,kw,kf. (750732)

58 ((care or health care or health-care or healthcare) adj (educat* or communicat* or inform* or instruct*)).tw,kw,kf. (33880)

59 exp Patient Education as Topic/ (217961)

60 ((caregiver? or care giver? or client? or family or families or patient? or person$2 or personally or user?) adj3 (communicat* or educat* or inform* or instruct* or resourc* or teach*)).tw,kw,kf. (682441)

61 exp Patient-Centered Care/ (960653)

62 ((patient-centered or patient-centred or patient-focus?ed) adj3 (approach or approaches or care or healthcare or health care or model or models or rehab*)).tw,kw,kf. (41278)

63 ((client-centered or client-centred or client-focus?ed) adj3 (approach or approaches or care or healthcare or health care or model or models or rehab*)).tw,kw,kf. (2493)

64 Patient Participation/ (64885)

65 ((caregiver* or care giver? or client* or family or families or patient* or person$2 or personally or user?) adj3 (activat* or engag* or empower* or involv* or participat*)).tw,kw,kf. (555808)

66 Self Care/ (111392)

67 Self-Management/ (70456)

68 ((person* or self) adj3 (manag* or care)).tw,kw,kf. (267090)

69 Aftercare/ (22704)

70 aftercare.tw,kw,kf. (12738)

71 ((after or followup or follow-up) adj (care or hospital* or treatment)).tw,kw,kf. (598299)

72 Patient Discharge/ (170974)

73 ((client* or patient or patients or facility or facilities or hospital or hospitals) adj3 discharg*).tw,kw,kf. (307568)

74 Continuity of Patient Care/ (321421)

75 Patient Transfer/ (40811)

76 Transitional Care/ (5556)

77 ((continuit* or continuum or path or paths or pathway* or transition*) adj (care or health care or health-care or healthcare)).tw,kw,kf. (7797)

78 Professional-Patient Relations/ (40441)

79 Nurse-Patient Relations/ (69318)

80 Physician-Patient Relations/ (83812)

81 (((professional* or clinician* or doctor* or nurse or nurses* or physician* or practitioner* or therapist*) adj3 (caregiver* or care giver? or client* or family or families or patient or patients)) and relations*).tw,kw,kf. (112158)

82 Therapeutic Alliance/ (7185)

83 therapeutic alliance?.tw,kw,kf. (15000)

84 or/20-83 [HEALTH CARE DELIVERY, REHAB, TRANSITIONAL CARE, ETC] (8130641)

85 Biomedical Technology/ (42388)

86 (technolog* adj3 (care or health or health care or health-care or healthcare)).tw,kw,kf. (69710)

87 exp Cell Phone/ (68746)

88 "Cell Phone Use"/ (1725)

89 exp Computers/ (303577)

90 Digital Technology/ (3384)

91 Electronic Mail/ (37013)

92 Internet/ (230792)

93 Internet Access/ (1600)

94 "Internet Use"/ (844)

95 Mobile Applications/ (29247)

96 Telecommunications/ (34540)

97 Telephone/ (58350)

98 exp Videoconferencing/ (10572)

99 (internet* or app or apps or computer* or cyber* or digital* or e-application? or e-mail* or email* or electronic mail* or iphone? or i-phone? or (mobile adj2 application?) or mobile-based or mobile phone? or online or (patient? adj3 portal*) or instant* messag* or (secure* adj3 communicat*) or (secure* adj3 messag*) or (secure* adj3 platform*) or (secure* adj3 portal*) or smarthome* or smart-home* or smarthub? or smart hub? or smartphone* or smart phone* or technolog* or telecommunicat* or tele-communicat* or telephon* or textmessag* or text-messag* or video* or web or webbased or web-based or webdeliver* or web-deliver* or "web 2.0").tw,kw,kf. (4310892)

100 or/85-99 [DIGITAL TECHNOLOGY] (4535913)

101 84 and 100 [DIGITAL CARE, PT 2] (902340)

102 19 or 101 [DIGITAL CARE, PTS 1-2] (2753132)

103 3 and 102 [HIP FRACTURES - DIGITAL CARE] (4667)

104 exp Animals/ not Humans/ (18241117)

105 103 not 104 [ANIMAL-ONLY REMOVED] (3803)

106 (Adolescent/ or exp Child/ or exp Infant/) not exp Adult/ (4791680)

107 105 not 106 [ADOLESCENT-, CHILD-, INFANT-ONLY REMOVED] (3774)

108 107 use medall [MEDLINE RECORDS] (1436)

109 exp hip fracture/ (74849)

110 ((hip or hips or femoral neck or (femur? adj2 neck?) or acetabul* or intertrochanter* or inter-trochanter* or pertrochanter* or per-trochanter* or subtrochanter* or sub-trochanter* or trochanter*) adj3 (break* or broke* or fractur*)).tw,kw,kf. (93947)

111 or/109-110 [HIP FRACTURES] (111241)

112 exp telemedicine/ (114712)

113 (telemed* or tele-med* or telecare or tele-care or teleconsult* or tele-consult* or teleconferenc* or tele-conferenc* or telecounsel* or tele-counsel* or telehealth* or tele-health* or telemonitor* or tele-monitor* or telepsychiatr* or tele-psychiatr* or telepsycholog* or tele-psycholog* or telerehab* or tele-rehab* or telesupport* or tele-support* or teletherap* or tele-therap* or teletreatment* or tele-treatment*).tw,kw,kf. (98347)

114 (ehealth* or e-health* or mhealth* or m-health* or emental health* or e-mental health* or mmental health* or m-mental health* or epsychiatr* or e-psychiatr* or mpsychiatr* or m-psychiatr* or epsychol* or e-psychol* or mpsychol* or m-psychol* or erehab* or e-rehab* or mrehab* or m-rehab* or etherap* or e-therap* or esupport* or e-support* or msupport* or m-support* or evisit* or e-visit* or mvisit* or m-visit*).tw,kw,kf. (44970)

115 (ecoach* or e-coach*).tw,kw,kf. (368)

116 (emedicine* or e-medicine*).tw,kw,kf. (296)

117 (mobile health* or mobile care or mobile counsel* or mobile medicine or mobile psychiatr* or mobile psycholog*).tw,kw,kf. (17596)

118 ((digital* or virtual* or remote*) adj3 (care or health* or healthcare or health-care)).tw,kw,kf. (34640)

119 ((digital* or virtual* or remote*) adj3 (appointment* or clinic or clinics or coach* or communicat* or conferenc* or consult* or followup or follow-up or hub or hubs or interven* or manag* or meet* or monitor* or rehab* or support* or therap* or tool or tools or treatment? or visit*)).tw,kw,kf. (96038)

120 (e-provider? or e-clinician? or e-counsel?or? or e-doctor? or e-nurse? or e-physician? or e-practitioner? or e-therapist? or m-provider? or m-clinician? or m-counsel?or? or m-doctor? or m-nurse? or m-physician? or m-practitioner? or m-therapist?).tw,kw,kf. (373)

121 (mobile provider? or mobile clinician? or mobile counsel?or? or mobile doctor? or mobile nurse? or mobile physician? or mobile practitioner? or mobile therapist?).tw,kw,kf. (176)

122 web-based intervention/ (3835)

123 exp computer assisted therapy/ (73812)

124 ((internet* or app or apps or computer* or cyber* or e-application? or e-mail* or email* or electronic mail* or iphone? or i-phone? or (mobile adj2 application?) or mobile-based or mobile phone? or online or (patient? adj3 portal*) or instant* messag* or (secure* adj3 communicat*) or (secure* adj3 messag*) or (secure* adj3 platform*) or (secure* adj3 portal*) or service or services or smarthome* or smart-home* or smarthub? or smart-hub? or smartphone* or smart phone* or technolog* or telecommunicat* or tele-communicat* or telephon* or textmessag* or text-messag* or video* or web or webbased or web-based or webdeliver* or web-deliver* or "web 2.0") adj3 (care or health care or health-care or healthcare or appointment* or coach* or conferenc* or consult* or interven* or manag* or meet* or monitor* or rehab* or support* or therap* or treatment? or visit*)).tw,kw,kf. (707884)

125 (internet* or app or apps or computer* or cyber* or e-application? or e-mail* or email* or electronic mail* or iphone? or i-phone? or (mobile adj2 application?) or mobile-based or mobile phone? or online or (patient? adj3 portal*) or instant* messag* or (secure* adj3 communicat*) or (secure* adj3 messag*) or (secure* adj3 platform*) or (secure* adj3 portal*) or smarthome* or smart-home* or smarthub? or smart hub? or smartphone* or smart phone* or technolog* or telecommunicat* or tele-communicat* or telephon* or textmessag* or text-messag* or video* or web or webbased or web-based or webdeliver* or web-deliver* or "web 2.0").ti,kw,kf. (1010965)

126 (internet* or app or apps or computer* or cyber* or e-application? or e-mail* or email* or electronic mail* or iphone? or i-phone? or (mobile adj2 application?) or mobile-based or mobile phone? or online or (patient? adj3 portal*) or instant* messag* or (secure* adj3 communicat*) or (secure* adj3 messag*) or (secure* adj3 platform*) or (secure* adj3 portal*) or smarthome* or smart-home* or smarthub? or smart hub? or smartphone* or smart phone* or technolog* or telecommunicat* or tele-communicat* or telephon* or textmessag* or text-messag* or video* or web or webbased or web-based or webdeliver* or web-deliver* or "web 2.0").ab. /freq=2 (1141672)

127 or/112-126 [DIGITAL CARE, PT 1] (2342542)

128 exp hip fracture/pc, rh, su, th [Prevention, Rehabilitation, Surgery, Therapy] (32838)

129 health care delivery/ (327843)

130 exp *health care delivery/ (1562320)

131 integrated health care system/ (26553)

132 ((deliver* or model or models or provid* or provision) adj3 (care or health care or health-care or healthcare)).ti,kw,kf. (100050)

133 health service/ (204643)

134 community care/ (63194)

135 community health nursing/ (46993)

136 rural health care/ (14408)

137 (healthservice* or health service* or healthcare service? or health care service?).ti,kw,kf. (143922)

138 exp geriatric nursing/ (27077)

139 home care/ (113318)

140 ((geriatric* or home) adj3 (care or health care or health-care or healthcare)).tw,kw,kf. (114734)

141 ((after operati* or after surger* or postoperati* or post-operati* or postsurg* or post-surg*) adj3 (care or health care or health-care or healthcare or followup or follow-up or rehab* or therap* or treatment*)).tw,kw,kf. (212983)

142 rehabilitation/ (147097)

143 home rehabilitation/ (956)

144 community based rehabilitation/ (877)

145 rehabilitation nursing/ (3056)

146 telerehabilitation/ (2905)

147 exp kinesiotherapy/ (95364)

148 ((exercis* or remedial* or rehab*) adj3 therap*).tw,kw,kf. (70823)

149 rh.fs. [Rehabilitation - Floating Subheading] (394686)

150 rehab*.ti,kw,kf. (282667)

151 rehab*.ab. /freq=2 (194601)

152 caregiver/ (144412)

153 family/ (242244)

154 exp ambulatory care/ (121572)

155 (ambulatory adj3 (care or health care or health-care or healthcare)).tw,kw,kf. (40052)

156 hospital patient/ (210028)

157 aged hospital patient/ (1212)

158 rehabilitation patient/ (1576)

159 inpatient*.tw,kw,kf. (419073)

160 outpatient/ (169281)

161 (outpatient* or out patient*).tw,kw,kf. (689770)

162 interpersonal communication/ (198669)

163 communicat*.ti,kw,kf. (269927)

164 communicat*.ab. /freq=2 (271887)

165 (miscommunicat* or mis-communicat*).tw,kw,kf. (3545)

166 (misunderstand* or mis-understand*).tw,kw,kf. (22723)

167 (misinform* or mis-inform*).tw,kw,kf. (13853)

168 information seeking/ (8412)

169 ((communicat* or provid* or provision* or seek* or search* or shar* or sought) adj3 information).tw,kw,kf. (750732)

170 ((care or health care or health-care or healthcare) adj (educat* or communicat* or inform* or instruct*)).tw,kw,kf. (33880)

171 patient education/ (221970)

172 ((caregiver? or care giver? or client? or family or families or patient? or person$2 or personally or user?) adj3 (communicat* or educat* or inform* or instruct* or resourc* or teach*)).tw,kw,kf. (682441)

173 patient care/ (345251)

174 ((patient-centered or patient-centred or patient-focus?ed) adj3 (approach or approaches or care or healthcare or health care or model or models or rehab*)).tw,kw,kf. (41278)

175 ((client-centered or client-centred or client-focus?ed) adj3 (approach or approaches or care or healthcare or health care or model or models or rehab*)).tw,kw,kf. (2493)

176 patient engagement/ (31169)

177 patient participation/ (64885)

178 ((caregiver* or care giver? or client* or family or families or patient* or person$2 or personally or user?) adj3 (activat* or engag* or empower* or involv* or participat*)).tw,kw,kf. (555808)

179 exp self care/ (163839)

180 ((person* or self) adj3 (manag* or care)).tw,kw,kf. (267090)

181 exp aftercare/ (2128947)

182 aftercare.tw,kw,kf. (12738)

183 ((after or followup or follow-up) adj (care or hospital* or treatment)).tw,kw,kf. (598299)

184 hospital discharge/ (159779)

185 ((client* or patient or patients or facility or facilities or hospital or hospitals) adj3 discharg*).tw,kw,kf. (307568)

186 transitional care/ (5556)

187 ((continuit* or continuum or path or paths or pathway* or transition*) adj (care or health care or health-care or healthcare)).tw,kw,kf. (7797)

188 professional-patient relationship/ (40827)

189 doctor patient relationship/ (6838)

190 nurse patient relationship/ (71399)

191 (((professional* or clinician* or doctor* or nurse or nurses* or physician* or practitioner* or therapist*) adj3 (caregiver* or care giver? or client* or family or families or patient or patients)) and relations*).tw,kw,kf. (112158)

192 therapeutic alliance/ (7185)

193 therapeutic alliance?.tw,kw,kf. (15000)

194 or/128-193 [HEALTH CARE DELIVERY, REHAB, TRANSITIONAL CARE, ETC] (9306904)

195 exp medical technology/ (36895)

196 (technolog* adj3 (care or health or health care or health-care or healthcare)).tw,kw,kf. (69710)

197 exp mobile phone/ (68746)

198 "cell phone use"/ (1725)

199 exp computer/ (256247)

200 digital technology/ (3384)

201 e-mail/ (30654)

202 internet/ (230792)

203 internet access/ (1600)

204 "internet use"/ (844)

205 exp mobile application/ (32717)

206 telecommunication/ (34540)

207 telephone/ (58350)

208 videoconferencing/ (10140)

209 (internet* or app or apps or computer* or cyber* or digital* or e-application? or e-mail* or email* or electronic mail* or iphone? or i-phone? or (mobile adj2 application?) or mobile-based or mobile phone? or online or (patient? adj3 portal*) or instant* messag* or (secure* adj3 communicat*) or (secure* adj3 messag*) or (secure* adj3 platform*) or (secure* adj3 portal*) or smarthome* or smart-home* or smarthub? or smart hub? or smartphone* or smart phone* or technolog* or telecommunicat* or tele-communicat* or telephon* or textmessag* or text-messag* or video* or web or webbased or web-based or webdeliver* or web-deliver* or "web 2.0").tw,kw,kf. (4310892)

210 or/195-209 [DIGITAL TECHNOLOGY] (4524427)

211 194 and 210 [DIGITAL CARE, PT 2] (958289)

212 127 or 211 [DIGITAL CARE, PTS 1-2] (2793400)

213 111 and 212 [HIP FRACTURES - DIGITAL CARE] (4833)

214 exp animal/ or exp animal experimentation/ or exp animal model/ or exp animal experiment/ or nonhuman/ or exp vertebrate/ (59148942)

215 exp human/ or exp human experimentation/ or exp human experiment/ (46084150)

216 214 not 215 (13066727)

217 213 not 216 [ANIMAL-ONLY REMOVED] (4810)

218 (juvenile/ or exp adolescent/ or exp child/) not (adult/ or middle aged/ or exp aged/) (4385475)

219 217 not 218 [ADOLESCENT-, CHILD-, INFANT-ONLY REMOVED] (4767)

220 219 use emczd [EMBASE RECORDS] (2595)

221 ((hip or hips or femoral neck or (femur? adj2 neck?) or acetabul* or intertrochanter* or inter-trochanter* or pertrochanter* or per-trochanter* or subtrochanter* or sub-trochanter* or trochanter*) adj3 (break* or broke* or fractur*)).tw. [HIP FRACTURES] (90972)

222 exp Telemedicine/ (114712)

223 (telemed* or tele-med* or telecare or tele-care or teleconsult* or tele-consult* or teleconferenc* or tele-conferenc* or telecounsel* or tele-counsel* or telehealth* or tele-health* or telemonitor* or tele-monitor* or telepsychiatr* or tele-psychiatr* or telepsycholog* or tele-psycholog* or telerehab* or tele-rehab* or telesupport* or tele-support* or teletherap* or tele-therap* or teletreatment* or tele-treatment*).tw. (85834)

224 Mobile Health/ (38006)

225 (ehealth* or e-health* or mhealth* or m-health* or emental health* or e-mental health* or mmental health* or m-mental health* or epsychiatr* or e-psychiatr* or mpsychiatr* or m-psychiatr* or epsychol* or e-psychol* or mpsychol* or m-psychol* or erehab* or e-rehab* or mrehab* or m-rehab* or etherap* or e-therap* or esupport* or e-support* or msupport* or m-support* or evisit* or e-visit* or mvisit* or m-visit*).tw. (34028)

226 (ecoach* or e-coach*).tw. (356)

227 (emedicine* or e-medicine*).tw. (257)

228 (mobile health* or mobile care or mobile counsel* or mobile medicine or mobile psychiatr* or mobile psycholog*).tw. (14697)

229 ((digital* or virtual* or remote*) adj3 (care or health* or healthcare or health-care)).tw. (29644)

230 ((digital* or virtual* or remote*) adj3 (appointment* or clinic or clinics or coach* or communicat* or conferenc* or consult* or followup or follow-up or hub or hubs or interven* or manag* or meet* or monitor* or rehab* or support* or therap* or tool or tools or treatment? or visit*)).tw. (90568)

231 (e-provider? or e-clinician? or e-counsel?or? or e-doctor? or e-nurse? or e-physician? or e-practitioner? or e-therapist? or m-provider? or m-clinician? or m-counsel?or? or m-doctor? or m-nurse? or m-physician? or m-practitioner? or m-therapist?).tw. (369)

232 (mobile provider? or mobile clinician? or mobile counsel?or? or mobile doctor? or mobile nurse? or mobile physician? or mobile practitioner? or mobile therapist?).tw. (174)

233 ((internet* or app or apps or computer* or cyber* or e-application? or e-mail* or email* or electronic mail* or iphone? or i-phone? or (mobile adj2 application?) or mobile-based or mobile phone? or online or (patient? adj3 portal*) or instant* messag* or (secure* adj3 communicat*) or (secure* adj3 messag*) or (secure* adj3 platform*) or (secure* adj3 portal*) or service or services or smarthome* or smart-home* or smarthub? or smart-hub? or smartphone* or smart phone* or technolog* or telecommunicat* or tele-communicat* or telephon* or textmessag* or text-messag* or video* or web or webbased or web-based or webdeliver* or web-deliver* or "web 2.0") adj3 (care or health care or health-care or healthcare or appointment* or coach* or conferenc* or consult* or interven* or manag* or meet* or monitor* or rehab* or support* or therap* or treatment? or visit*)).tw. (690747)

234 (internet* or app or apps or computer* or cyber* or e-application? or e-mail* or email* or electronic mail* or iphone? or i-phone? or (mobile adj2 application?) or mobile-based or mobile phone? or online or (patient? adj3 portal*) or instant* messag* or (secure* adj3 communicat*) or (secure* adj3 messag*) or (secure* adj3 platform*) or (secure* adj3 portal*) or smarthome* or smart-home* or smarthub? or smart hub? or smartphone* or smart phone* or technolog* or telecommunicat* or tele-communicat* or telephon* or textmessag* or text-messag* or video* or web or webbased or web-based or webdeliver* or web-deliver* or "web 2.0").ti,id. (897720)

235 (internet* or app or apps or computer* or cyber* or e-application? or e-mail* or email* or electronic mail* or iphone? or i-phone? or (mobile adj2 application?) or mobile-based or mobile phone? or online or (patient? adj3 portal*) or instant* messag* or (secure* adj3 communicat*) or (secure* adj3 messag*) or (secure* adj3 platform*) or (secure* adj3 portal*) or smarthome* or smart-home* or smarthub? or smart hub? or smartphone* or smart phone* or technolog* or telecommunicat* or tele-communicat* or telephon* or textmessag* or text-messag* or video* or web or webbased or web-based or webdeliver* or web-deliver* or "web 2.0").ab. /freq=2 (1141672)

236 or/222-235 [DIGITAL CARE, PT 1] (2194696)

237 Health Care Delivery/ (327843)

238 Integrated Services/ (4640)

239 ((deliver* or model or models or provid* or provision) adj3 (care or health care or health-care or healthcare)).ti,id. (84673)

240 Health Care Services/ (47281)

241 Rural Health/ (26811)

242 (healthservice* or health service* or healthcare service? or health care service?).ti,id. (116698)

243 Home Care/ (113318)

244 ((geriatric* or home) adj3 (care or health care or health-care or healthcare)).tw. (109746)

245 ((after operati* or after surger* or postoperati* or post-operati* or postsurg* or post-surg*) adj3 (care or health care or health-care or healthcare or followup or follow-up or rehab* or therap* or treatment*)).tw. (201320)

246 Rehabilitation/ (147097)

247 Telerehabilitation/ (2905)

248 Physical Therapy/ (148802)

249 ((exercis* or remedial* or rehab*) adj3 therap*).tw. (61579)

250 rehab*.ti,id. (230030)

251 rehab*.ab. /freq=2 (194601)

252 Caregivers/ (158043)

253 Caregiving/ (11184)

254 Family/ (242244)

255 Elder Care/ (5605)

256 Outpatient Treatment/ (7403)

257 (ambulatory adj3 (care or health care or health-care or healthcare)).tw. (36852)

258 Patients/ (1528137)

259 Geriatric Patients/ (13965)

260 Hospitalized Patients/ (223918)

261 inpatient*.tw. (416282)

262 Outpatients/ (160456)

263 (outpatient* or out patient*).tw. (685050)

264 Surgical Patients/ (3233)

265 Communication/ (270458)

266 communicat*.ti,id. (263918)

267 communicat*.ab. /freq=2 (271887)

268 (miscommunicat* or mis-communicat*).tw. (3523)

269 (misunderstand* or mis-understand*).tw. (22699)

270 (misinform* or mis-inform*).tw. (13384)

271 Information Seeking/ (8412)

272 ((communicat* or provid* or provision* or seek* or search* or shar* or sought) adj3 information).tw. (749048)

273 ((care or health care or health-care or healthcare) adj (educat* or communicat* or inform* or instruct*)).tw. (31695)

274 Client Education/ (4370)

275 ((caregiver? or care giver? or client? or family or families or patient? or person$2 or personally or user?) adj3 (communicat* or educat* or inform* or instruct* or resourc* or teach*)).tw. (667583)

276 Patient Centered Care/ (242927)

277 ((patient-centered or patient-centred or patient-focus?ed) adj3 (approach or approaches or care or healthcare or health care or model or models or rehab*)).tw. (35616)

278 ((client-centered or client-centred or client-focus?ed) adj3 (approach or approaches or care or healthcare or health care or model or models or rehab*)).tw. (2438)

279 Client Participation/ (2788)

280 ((caregiver* or care giver? or client* or family or families or patient* or person$2 or personally or user?) adj3 (activat* or engag* or empower* or involv* or participat*)).tw. (550540)

281 Self-Care/ (111392)

282 exp Self-Management/ (107465)

283 ((person* or self) adj3 (manag* or care)).tw. (255672)

284 Aftercare/ (22704)

285 aftercare.tw. (12215)

286 ((after or followup or follow-up) adj (care or hospital* or treatment)).tw. (594462)

287 Discharge Planning/ (195771)

288 Hospital Discharge/ (159779)

289 ((client* or patient or patients or facility or facilities or hospital or hospitals) adj3 discharg*).tw. (305030)

290 Continuum of Care/ (23136)

291 Client Transfer/ (285)

292 ((continuit* or continuum or path or paths or pathway* or transition*) adj (care or health care or health-care or healthcare)).tw. (6955)

293 (((professional* or clinician* or doctor* or nurse or nurses* or physician* or practitioner* or therapist*) adj3 (caregiver* or care giver? or client* or family or families or patient or patients)) and relations*).tw. (91085)

294 Therapeutic Alliance/ (7185)

295 therapeutic alliance?.tw. (14539)

296 or/237-295 [HEALTH CARE DELIVERY, REHAB, TRANSITIONAL CARE, ETC] (7433349)

297 (technolog* adj3 (care or health or health care or health-care or healthcare)).tw. (65697)

298 exp Mobile Phones/ (29320)

299 Mobile Devices/ (7152)

300 exp Computers/ (303577)

301 Digital Health Resources/ (63)

302 Digital Technology/ (3384)

303 Computer Mediated Communication/ (6368)

304 Internet/ (230792)

305 Internet Usage/ (3660)

306 exp Computer Applications/ (81596)

307 Mobile Applications/ (29247)

308 "Information and Communication Technology"/ (10416)

309 Telephone Systems/ (2304)

310 exp Teleconferencing/ (3650)

311 (internet* or app or apps or computer* or cyber* or digital* or e-application? or e-mail* or email* or electronic mail* or iphone? or i-phone? or (mobile adj2 application?) or mobile-based or mobile phone? or online or (patient? adj3 portal*) or instant* messag* or (secure* adj3 communicat*) or (secure* adj3 messag*) or (secure* adj3 platform*) or (secure* adj3 portal*) or smarthome* or smart-home* or smarthub? or smart hub? or smartphone* or smart phone* or technolog* or telecommunicat* or tele-communicat* or telephon* or textmessag* or text-messag* or video* or web or webbased or web-based or webdeliver* or web-deliver* or "web 2.0").tw. (4234398)

312 or/297-311 [DIGITAL TECHNOLOGY] (4422601)

313 296 and 312 [DIGITAL CARE, PT 2] (776306)

314 236 or 313 [DIGITAL CARE, PTS 1-2] (2566479)

315 221 and 314 [HIP FRACTURES - DIGITAL CARE] (3464)

316 315 use medall,emczd,coch,cctr,dare (3372)

317 315 not 316 [PSYCINFO RECORDS] (92)

318 exp Hip Fractures/ (74849)

319 ((hip or hips or femoral neck or (femur? adj2 neck?) or acetabul* or intertrochanter* or inter-trochanter* or pertrochanter* or per-trochanter* or subtrochanter* or sub-trochanter* or trochanter*) adj3 (break* or broke* or fractur*)).ti,ab,kw. (91718)

320 or/318-319 [HIP FRACTURES] (110248)

321 exp Telemedicine/ (114712)

322 (telemed* or tele-med* or telecare or tele-care or teleconsult* or tele-consult* or teleconferenc* or tele-conferenc* or telecounsel* or tele-counsel* or telehealth* or tele-health* or telemonitor* or tele-monitor* or telepsychiatr* or tele-psychiatr* or telepsycholog* or tele-psycholog* or telerehab* or tele-rehab* or telesupport* or tele-support* or teletherap* or tele-therap* or teletreatment* or tele-treatment*).ti,ab,kw. (96337)

323 (ehealth* or e-health* or mhealth* or m-health* or emental health* or e-mental health* or mmental health* or m-mental health* or epsychiatr* or e-psychiatr* or mpsychiatr* or m-psychiatr* or epsychol* or e-psychol* or mpsychol* or m-psychol* or erehab* or e-rehab* or mrehab* or m-rehab* or etherap* or e-therap* or esupport* or e-support* or msupport* or m-support* or evisit* or e-visit* or mvisit* or m-visit*).ti,ab,kw. (42794)

324 (ecoach* or e-coach*).ti,ab,kw. (359)

325 (emedicine* or e-medicine*).ti,ab,kw. (289)

326 (mobile health* or mobile care or mobile counsel* or mobile medicine or mobile psychiatr* or mobile psycholog*).ti,ab,kw. (17110)

327 ((digital* or virtual* or remote*) adj3 (care or health* or healthcare or health-care)).ti,ab,kw. (31360)

328 ((digital* or virtual* or remote*) adj3 (appointment* or clinic or clinics or coach* or communicat* or conferenc* or consult* or followup or follow-up or hub or hubs or interven* or manag* or meet* or monitor* or rehab* or support* or therap* or tool or tools or treatment? or visit*)).ti,ab,kw. (92492)

329 (e-provider? or e-clinician? or e-counsel?or? or e-doctor? or e-nurse? or e-physician? or e-practitioner? or e-therapist? or m-provider? or m-clinician? or m-counsel?or? or m-doctor? or m-nurse? or m-physician? or m-practitioner? or m-therapist?).ti,ab,kw. (358)

330 (mobile provider? or mobile clinician? or mobile counsel?or? or mobile doctor? or mobile nurse? or mobile physician? or mobile practitioner? or mobile therapist?).ti,ab,kw. (174)

331 Internet-Based Intervention/ (2113)

332 exp Therapy, Computer-Assisted/ (61754)

333 ((internet* or app or apps or computer* or cyber* or e-application? or e-mail* or email* or electronic mail* or iphone? or i-phone? or (mobile adj2 application?) or mobile-based or mobile phone? or online or (patient? adj3 portal*) or instant* messag* or (secure* adj3 communicat*) or (secure* adj3 messag*) or (secure* adj3 platform*) or (secure* adj3 portal*) or service or services or smarthome* or smart-home* or smarthub? or smart-hub? or smartphone* or smart phone* or technolog* or telecommunicat* or tele-communicat* or telephon* or textmessag* or text-messag* or video* or web or webbased or web-based or webdeliver* or web-deliver* or "web 2.0") adj3 (care or health care or health-care or healthcare or appointment* or coach* or conferenc* or consult* or interven* or manag* or meet* or monitor* or rehab* or support* or therap* or treatment? or visit*)).ti,ab,kw. (685616)

334 (internet* or app or apps or computer* or cyber* or e-application? or e-mail* or email* or electronic mail* or iphone? or i-phone? or (mobile adj2 application?) or mobile-based or mobile phone? or online or (patient? adj3 portal*) or instant* messag* or (secure* adj3 communicat*) or (secure* adj3 messag*) or (secure* adj3 platform*) or (secure* adj3 portal*) or smarthome* or smart-home* or smarthub? or smart hub? or smartphone* or smart phone* or technolog* or telecommunicat* or tele-communicat* or telephon* or textmessag* or text-messag* or video* or web or webbased or web-based or webdeliver* or web-deliver* or "web 2.0").ti. (836393)

335 (internet* or app or apps or computer* or cyber* or e-application? or e-mail* or email* or electronic mail* or iphone? or i-phone? or (mobile adj2 application?) or mobile-based or mobile phone? or online or (patient? adj3 portal*) or instant* messag* or (secure* adj3 communicat*) or (secure* adj3 messag*) or (secure* adj3 platform*) or (secure* adj3 portal*) or smarthome* or smart-home* or smarthub? or smart hub? or smartphone* or smart phone* or technolog* or telecommunicat* or tele-communicat* or telephon* or textmessag* or text-messag* or video* or web or webbased or web-based or webdeliver* or web-deliver* or "web 2.0").ab. /freq=2 (1141672)

336 or/321-335 [DIGITAL CARE, PT 1] (2212734)

337 exp Hip Fractures/pc, rh, su, th [Prevention, Rehabilitation, Surgery, Therapy] (32838)

338 Delivery of Health Care/ (269689)

339 exp *Delivery of Health Care/ (1485247)

340 Delivery of Health Care, Integrated/ (25449)

341 ((deliver* or model or models or provid* or provision) adj3 (care or health care or health-care or healthcare)).ti. (76383)

342 Health Services/ (197647)

343 Community Health Services/ (89281)

344 Rural Health Services/ (27382)

345 Suburban Health Services/ (177602)

346 Urban Health Services/ (161267)

347 (healthservice* or health service* or healthcare service? or health care service?).ti. (91078)

348 Geriatric Nursing/ (27030)

349 Home Nursing/ (75362)

350 ((geriatric* or home) adj3 (care or health care or health-care or healthcare)).ti,ab,kw. (109837)

351 ((after operati* or after surger* or postoperati* or post-operati* or postsurg* or post-surg*) adj3 (care or health care or health-care or healthcare or followup or follow-up or rehab* or therap* or treatment*)).ti,ab,kw. (207270)

352 Rehabilitation/ (147097)

353 Rehabilitation Nursing/ (3056)

354 exp Exercise Therapy/ (170873)

355 ((exercis* or remedial* or rehab*) adj3 therap*).ti,ab,kw. (65918)

356 rh.fs. [Rehabilitation - Floating Subheading] (394686)

357 rehab*.ti. (211346)

358 rehab*.ab. /freq=2 (194601)

359 Caregivers/ (158043)

360 Family/ (242244)

361 Ambulatory Care/ (98887)

362 (ambulatory adj3 (care or health care or health-care or healthcare)).ti,ab,kw. (37187)

363 Inpatients/ (209614)

364 inpatient*.ti,ab,kw. (414263)

365 Outpatients/ (160456)

366 (outpatient* or out patient*).ti,ab,kw. (676356)

367 Communication/ (270458)

368 communicat*.ti. (214490)

369 communicat*.ab. /freq=2 (271887)

370 (miscommunicat* or mis-communicat*).ti,ab,kw. (3507)

371 (misunderstand* or mis-understand*).ti,ab,kw. (22523)

372 (misinform* or mis-inform*).ti,ab,kw. (13649)

373 Information Seeking Behavior/ (7412)

374 ((communicat* or provid* or provision* or seek* or search* or shar* or sought) adj3 information).ti,ab,kw. (740991)

375 ((care or health care or health-care or healthcare) adj (educat* or communicat* or inform* or instruct*)).ti,ab,kw. (32434)

376 exp Patient Education as Topic/ (217961)

377 ((caregiver? or care giver? or client? or family or families or patient? or person$2 or personally or user?) adj3 (communicat* or educat* or inform* or instruct* or resourc* or teach*)).ti,ab,kw. (665924)

378 exp Patient-Centered Care/ (960653)

379 ((patient-centered or patient-centred or patient-focus?ed) adj3 (approach or approaches or care or healthcare or health care or model or models or rehab*)).ti,ab,kw. (34938)

380 ((client-centered or client-centred or client-focus?ed) adj3 (approach or approaches or care or healthcare or health care or model or models or rehab*)).ti,ab,kw. (2380)

381 Patient Participation/ (64885)

382 ((caregiver* or care giver? or client* or family or families or patient* or person$2 or personally or user?) adj3 (activat* or engag* or empower* or involv* or participat*)).ti,ab,kw. (547685)

383 Self Care/ (111392)

384 Self-Management/ (70456)

385 ((person* or self) adj3 (manag* or care)).ti,ab,kw. (256216)

386 Aftercare/ (22704)

387 aftercare.ti,ab,kw. (12440)

388 ((after or followup or follow-up) adj (care or hospital* or treatment)).ti,ab,kw. (595840)

389 Patient Discharge/ (170974)

390 ((client* or patient or patients or facility or facilities or hospital or hospitals) adj3 discharg*).ti,ab,kw. (304725)

391 Continuity of Patient Care/ (321421)

392 Patient Transfer/ (40811)

393 Transitional Care/ (5556)

394 ((continuit* or continuum or path or paths or pathway* or transition*) adj (care or health care or health-care or healthcare)).ti,ab,kw. (7025)

395 Professional-Patient Relations/ (40441)

396 Nurse-Patient Relations/ (69318)

397 Physician-Patient Relations/ (83812)

398 (((professional* or clinician* or doctor* or nurse or nurses* or physician* or practitioner* or therapist*) adj3 (caregiver* or care giver? or client* or family or families or patient or patients)) and relations*).ti,ab,kw. (88418)

399 Therapeutic Alliance/ (7185)

400 therapeutic alliance?.ti,ab,kw. (14212)

401 or/337-400 [HEALTH CARE DELIVERY, REHAB, TRANSITIONAL CARE, ETC] (8006697)

402 Biomedical Technology/ (42388)

403 (technolog* adj3 (care or health or health care or health-care or healthcare)).ti,ab,kw. (64571)

404 exp Cell Phone/ (68746)

405 "Cell Phone Use"/ (1725)

406 exp Computers/ (303577)

407 Digital Technology/ (3384)

408 Electronic Mail/ (37013)

409 Internet/ (230792)

410 Internet Access/ (1600)

411 "Internet Use"/ (844)

412 Mobile Applications/ (29247)

413 Telecommunications/ (34540)

414 Telephone/ (58350)

415 exp Videoconferencing/ (10572)

416 (internet* or app or apps or computer* or cyber* or digital* or e-application? or e-mail* or email* or electronic mail* or iphone? or i-phone? or (mobile adj2 application?) or mobile-based or mobile phone? or online or (patient? adj3 portal*) or instant* messag* or (secure* adj3 communicat*) or (secure* adj3 messag*) or (secure* adj3 platform*) or (secure* adj3 portal*) or smarthome* or smart-home* or smarthub? or smart hub? or smartphone* or smart phone* or technolog* or telecommunicat* or tele-communicat* or telephon* or textmessag* or text-messag* or video* or web or webbased or web-based or webdeliver* or web-deliver* or "web 2.0").ti,ab,kw. (4255233)

417 or/402-416 [DIGITAL TECHNOLOGY] (4483386)

418 401 and 417 [DIGITAL CARE, PT 2] (868795)

419 336 or 418 [DIGITAL CARE, PTS 1-2] (2622298)

420 320 and 419 [HIP FRACTURES - DIGITAL CARE] (4249)

421 (Adolescent/ or exp Child/ or exp Infant/) not exp Adult/ (4791680)

422 420 not 421 [ADOLESCENT-, CHILD-, INFANT-ONLY REMOVED] (4213)

423 422 use coch,cctr [COCHRANE RECORDS] (343)

424 ((hip or hips or femoral neck or (femur? adj2 neck?) or acetabul* or intertrochanter* or inter-trochanter* or pertrochanter* or per-trochanter* or subtrochanter* or sub-trochanter* or trochanter*) adj3 (break* or broke* or fractur*)).tw,kw. [HIP FRACTURES] (91963)

425 (telemed* or tele-med* or telecare or tele-care or teleconsult* or tele-consult* or teleconferenc* or tele-conferenc* or telecounsel* or tele-counsel* or telehealth* or tele-health* or telemonitor* or tele-monitor* or telepsychiatr* or tele-psychiatr* or telepsycholog* or tele-psycholog* or telerehab* or tele-rehab* or telesupport* or tele-support* or teletherap* or tele-therap* or teletreatment* or tele-treatment*).tw,kw. (97709)

426 (ehealth* or e-health* or mhealth* or m-health* or emental health* or e-mental health* or mmental health* or m-mental health* or epsychiatr* or e-psychiatr* or mpsychiatr* or m-psychiatr* or epsychol* or e-psychol* or mpsychol* or m-psychol* or erehab* or e-rehab* or mrehab* or m-rehab* or etherap* or e-therap* or esupport* or e-support* or msupport* or m-support* or evisit* or e-visit* or mvisit* or m-visit*).tw,kw. (44371)

427 (ecoach* or e-coach*).tw,kw. (368)

428 (emedicine* or e-medicine*).tw,kw. (296)

429 (mobile health* or mobile care or mobile counsel* or mobile medicine or mobile psychiatr* or mobile psycholog*).tw,kw. (17528)

430 ((digital* or virtual* or remote*) adj3 (care or health* or healthcare or health-care)).tw,kw. (31844)

431 ((digital* or virtual* or remote*) adj3 (appointment* or clinic or clinics or coach* or communicat* or conferenc* or consult* or followup or follow-up or hub or hubs or interven* or manag* or meet* or monitor* or rehab* or support* or therap* or tool or tools or treatment? or visit*)).tw,kw. (93277)

432 (e-provider? or e-clinician? or e-counsel?or? or e-doctor? or e-nurse? or e-physician? or e-practitioner? or e-therapist? or m-provider? or m-clinician? or m-counsel?or? or m-doctor? or m-nurse? or m-physician? or m-practitioner? or m-therapist?).tw,kw. (373)

433 (mobile provider? or mobile clinician? or mobile counsel?or? or mobile doctor? or mobile nurse? or mobile physician? or mobile practitioner? or mobile therapist?).tw,kw. (175)

434 ((internet* or app or apps or computer* or cyber* or e-application? or e-mail* or email* or electronic mail* or iphone? or i-phone? or (mobile adj2 application?) or mobile-based or mobile phone? or online or (patient? adj3 portal*) or instant* messag* or (secure* adj3 communicat*) or (secure* adj3 messag*) or (secure* adj3 platform*) or (secure* adj3 portal*) or service or services or smarthome* or smart-home* or smarthub? or smart-hub? or smartphone* or smart phone* or technolog* or telecommunicat* or tele-communicat* or telephon* or textmessag* or text-messag* or video* or web or webbased or web-based or webdeliver* or web-deliver* or "web 2.0") adj3 (care or health care or health-care or healthcare or appointment* or coach* or conferenc* or consult* or interven* or manag* or meet* or monitor* or rehab* or support* or therap* or treatment? or visit*)).tw,kw. (700548)

435 (internet* or app or apps or computer* or cyber* or e-application? or e-mail* or email* or electronic mail* or iphone? or i-phone? or (mobile adj2 application?) or mobile-based or mobile phone? or online or (patient? adj3 portal*) or instant* messag* or (secure* adj3 communicat*) or (secure* adj3 messag*) or (secure* adj3 platform*) or (secure* adj3 portal*) or smarthome* or smart-home* or smarthub? or smart hub? or smartphone* or smart phone* or technolog* or telecommunicat* or tele-communicat* or telephon* or textmessag* or text-messag* or video* or web or webbased or web-based or webdeliver* or web-deliver* or "web 2.0").ti. (836393)

436 (internet* or app or apps or computer* or cyber* or e-application? or e-mail* or email* or electronic mail* or iphone? or i-phone? or (mobile adj2 application?) or mobile-based or mobile phone? or online or (patient? adj3 portal*) or instant* messag* or (secure* adj3 communicat*) or (secure* adj3 messag*) or (secure* adj3 platform*) or (secure* adj3 portal*) or smarthome* or smart-home* or smarthub? or smart hub? or smartphone* or smart phone* or technolog* or telecommunicat* or tele-communicat* or telephon* or textmessag* or text-messag* or video* or web or webbased or web-based or webdeliver* or web-deliver* or "web 2.0").tw. /freq=2 (1354289)

437 or/425-436 [DIGITAL CARE, PT 1] (2184695)

438 ((deliver* or model or models or provid* or provision) adj3 (care or health care or health-care or healthcare)).ti. (76383)

439 (healthservice* or health service* or healthcare service? or health care service?).ti. (91078)

440 ((geriatric* or home) adj3 (care or health care or health-care or healthcare)).tw,kw. (111777)

441 ((after operati* or after surger* or postoperati* or post-operati* or postsurg* or post-surg*) adj3 (care or health care or health-care or healthcare or followup or follow-up or rehab* or therap* or treatment*)).tw,kw. (208356)

442 ((exercis* or remedial* or rehab*) adj3 therap*).tw,kw. (67324)

443 rehab*.ti. (211346)

444 rehab*.tw. /freq=2 (243822)

445 (ambulatory adj3 (care or health care or health-care or healthcare)).tw,kw. (37495)

446 inpatient*.tw,kw. (418742)

447 (outpatient* or out patient*).tw,kw. (688809)

448 communicat*.ti. (214490)

449 communicat*.tw. /freq=2 (333826)

450 (miscommunicat* or mis-communicat*).tw,kw. (3545)

451 (misunderstand* or mis-understand*).tw,kw. (22720)

452 (misinform* or mis-inform*).tw,kw. (13831)

453 ((communicat* or provid* or provision* or seek* or search* or shar* or sought) adj3 information).tw,kw. (749509)

454 ((care or health care or health-care or healthcare) adj (educat* or communicat* or inform* or instruct*)).tw,kw. (32973)

455 ((caregiver? or care giver? or client? or family or families or patient? or person$2 or personally or user?) adj3 (communicat* or educat* or inform* or instruct* or resourc* or teach*)).tw,kw. (673884)

456 ((patient-centered or patient-centred or patient-focus?ed) adj3 (approach or approaches or care or healthcare or health care or model or models or rehab*)).tw,kw. (35725)

457 ((client-centered or client-centred or client-focus?ed) adj3 (approach or approaches or care or healthcare or health care or model or models or rehab*)).tw,kw. (2450)

458 ((caregiver* or care giver? or client* or family or families or patient* or person$2 or personally or user?) adj3 (activat* or engag* or empower* or involv* or participat*)).tw,kw. (552210)

459 ((person* or self) adj3 (manag* or care)).tw,kw. (261153)

460 aftercare.tw,kw. (12688)

461 ((after or followup or follow-up) adj (care or hospital* or treatment)).tw,kw. (597997)

462 ((client* or patient or patients or facility or facilities or hospital or hospitals) adj3 discharg*).tw,kw. (306687)

463 ((continuit* or continuum or path or paths or pathway* or transition*) adj (care or health care or health-care or healthcare)).tw,kw. (7164)

464 (((professional* or clinician* or doctor* or nurse or nurses* or physician* or practitioner* or therapist*) adj3 (caregiver* or care giver? or client* or family or families or patient or patients)) and relations*).tw,kw. (92210)

465 therapeutic alliance?.tw,kw. (14983)

466 or/438-465 [HEALTH CARE DELIVERY, REHAB, TRANSITIONAL CARE, ETC] (4854799)

467 (technolog* adj3 (care or health or health care or health-care or healthcare)).tw,kw. (66382)

468 (internet* or app or apps or computer* or cyber* or digital* or e-application? or e-mail* or email* or electronic mail* or iphone? or i-phone? or (mobile adj2 application?) or mobile-based or mobile phone? or online or (patient? adj3 portal*) or instant* messag* or (secure* adj3 communicat*) or (secure* adj3 messag*) or (secure* adj3 platform*) or (secure* adj3 portal*) or smarthome* or smart-home* or smarthub? or smart hub? or smartphone* or smart phone* or technolog* or telecommunicat* or tele-communicat* or telephon* or textmessag* or text-messag* or video* or web or webbased or web-based or webdeliver* or web-deliver* or "web 2.0").tw,kw. (4284309)

469 467 or 468 [DIGITAL TECHNOLOGY] (4284309)

470 466 and 469 [DIGITAL CARE, PT 2] (596179)

471 437 or 470 [DIGITAL CARE, PTS 1-2] (2448201)

472 424 and 471 [HIP FRACTURES - DIGITAL CARE] (3294)

473 472 use dare [DARE RECORDS] (45)

474 108 or 220 or 317 or 423 or 473 [ALL DATABASES] (4511)

475 remove duplicates from 474 (**3148**) [**TOTAL UNIQUE RECORDS**]

476 475 use medall [MEDLINE UNIQUE RECORDS] (1433)

477 475 use emczd [EMBASE UNIQUE RECORDS] (1456)

478 475 use medall,emczd,coch,cctr,dare (3120)

479 475 not 478 [PSYCINFO UNIQUE RECORDS] (28)

480 475 use cctr [CENTRAL UNIQUE RECORDS] (180)

481 475 use coch [CDSR UNIQUE RECORDS] (6)

482 475 use dare [DARE UNIQUE RECORDS] (45)

***************************

CINAHL

| # | Query | Limiters/Expanders | Results |
| --- | --- | --- | --- |
| S100 | S3 AND S98 | Limiters - Exclude MEDLINE records  Expanders - Apply related words; Apply equivalent subjects  Search modes - Boolean/Phrase | 733 |
| S99 | S3 AND S98 | Expanders - Apply related words; Apply equivalent subjects  Search modes - Boolean/Phrase | 1,691 |
| S98 | S19 OR S97 | Expanders - Apply related words; Apply equivalent subjects  Search modes - Boolean/Phrase | 551,799 |
| S97 | S83 AND S96 | Expanders - Apply related words; Apply equivalent subjects  Search modes - Boolean/Phrase | 228,431 |
| S96 | S84 OR S85 OR S86 OR S87 OR S88 OR S89 OR S90 OR S91 OR S92 OR S93 OR S94 OR S95 | Expanders - Apply related words; Apply equivalent subjects  Search modes - Boolean/Phrase | 1,114,270 |
| S95 | TI ( internet* or app or apps or computer* or cyber* or digital* or (e W0 application#) or (e W0 mail*) or email* or (electronic W0 mail*) or iphone# or (i W0 phone#) or (mobile N2 application#) or "mobile-based" or (mobile W0 phone#) or online or (patient# N3 portal*) or (instant* W0 messag*) or (secure* N3 communicat*) or (secure* N3 messag*) or (secure* N3 platform*) or (secure* N3 portal*) or smarthome* or (smart W0 home*) or smarthub# or (smart W0 hub#) or smartphone* or (smart W0 phone*) or technolog* or telecommunicat* or (tele W0 communicat*) or telephon* or textmessag* or (text W0 messag*) or video* or web or webbased or "web-based" or webdeliver* or (web W0 deliver*) or "web 2.0" ) OR AB ( internet* or app or apps or computer* or cyber* or digital* or (e W0 application#) or (e W0 mail*) or email* or (electronic W0 mail*) or iphone# or (i W0 phone#) or (mobile N2 application#) or "mobile-based" or (mobile W0 phone#) or online or (patient# N3 portal*) or (instant* W0 messag*) or (secure* N3 communicat*) or (secure* N3 messag*) or (secure* N3 platform*) or (secure* N3 portal*) or smarthome* or (smart W0 home*) or smarthub# or (smart W0 hub#) or smartphone* or (smart W0 phone*) or technolog* or telecommunicat* or (tele W0 communicat*) or telephon* or textmessag* or (text W0 messag*) or video* or web or webbased or "web-based" or webdeliver* or (web W0 deliver*) or "web 2.0" ) | Expanders - Apply related words; Apply equivalent subjects  Search modes - Boolean/Phrase | 486,398 |
| S94 | (MH "Videoconferencing+") OR (MH "Teleconferencing") | Expanders - Apply related words; Apply equivalent subjects  Search modes - Boolean/Phrase | 6,898 |
| S93 | (MH "Telephone") | Expanders - Apply related words; Apply equivalent subjects  Search modes - Boolean/Phrase | 17,490 |
| S92 | (MH "Telecommunications") | Expanders - Apply related words; Apply equivalent subjects  Search modes - Boolean/Phrase | 2,592 |
| S91 | (MH "Mobile Applications") | Expanders - Apply related words; Apply equivalent subjects  Search modes - Boolean/Phrase | 10,625 |
| S90 | (MH "Internet Access") | Expanders - Apply related words; Apply equivalent subjects  Search modes - Boolean/Phrase | 310 |
| S89 | (MH "Internet") | Expanders - Apply related words; Apply equivalent subjects  Search modes - Boolean/Phrase | 53,492 |
| S88 | (MH "Email") OR (MH "Instant Messaging") OR (MH "Text Messaging") | Expanders - Apply related words; Apply equivalent subjects  Search modes - Boolean/Phrase | 10,885 |
| S87 | (MH "Digital Technology") | Expanders - Apply related words; Apply equivalent subjects  Search modes - Boolean/Phrase | 999 |
| S86 | (MH "Computers and Computerization+") | Expanders - Apply related words; Apply equivalent subjects  Search modes - Boolean/Phrase | 798,125 |
| S85 | (MH "Cellular Phone+") | Expanders - Apply related words; Apply equivalent subjects  Search modes - Boolean/Phrase | 9,228 |
| S84 | TI ( technolog* N3 (care or health or "health care" or "health-care" or healthcare) ) OR AB ( technolog* N3 (care or health or "health care" or "health-care" or healthcare) ) | Expanders - Apply related words; Apply equivalent subjects  Search modes - Boolean/Phrase | 19,343 |
| S83 | S20 OR S21 OR S22 OR S23 OR S24 OR S25 OR S26 OR S27 OR S28 OR S29 OR S30 OR S31 OR S32 OR S33 OR S34 OR S35 OR S36 OR S37 OR S38 OR S39 OR S40 OR S41 OR S42 OR S43 OR S44 OR S45 OR S46 OR S47 OR S48 OR S49 OR S50 OR S51 OR S52 OR S53 OR S54 OR S55 OR S56 OR S57 OR S58 OR S59 OR S60 OR S61 OR S62 OR S63 OR S64 OR S65 OR S66 OR S67 OR S68 OR S69 OR S70 OR S71 OR S72 OR S73 OR S74 OR S75 OR S76 OR S77 OR S78 OR S79 OR S80 OR S81 OR S82 | Expanders - Apply related words; Apply equivalent subjects  Search modes - Boolean/Phrase | 1,130,899 |
| S82 | TI therapeutic W0 alliance# OR AB therapeutic W0 alliance# | Expanders - Apply related words; Apply equivalent subjects  Search modes - Boolean/Phrase | 1,673 |
| S81 | (MH "Therapeutic Alliance") | Expanders - Apply related words; Apply equivalent subjects  Search modes - Boolean/Phrase | 495 |
| S80 | TI ( ((professional* or clinician* or doctor* or nurse or nurses* or physician* or practitioner* or therapist*) N3 (caregiver* or (care W0 giver#) or client* or family or families or patient or patients)) and relations* ) OR AB ( ((professional* or clinician* or doctor* or nurse or nurses* or physician* or practitioner* or therapist*) N3 (caregiver* or (care W0 giver#) or client* or family or families or patient or patients)) and relations* ) | Expanders - Apply related words; Apply equivalent subjects  Search modes - Boolean/Phrase | 18,379 |
| S79 | (MH "Physician-Patient Relations") | Expanders - Apply related words; Apply equivalent subjects  Search modes - Boolean/Phrase | 34,940 |
| S78 | (MH "Nurse-Patient Relations") | Expanders - Apply related words; Apply equivalent subjects  Search modes - Boolean/Phrase | 28,519 |
| S77 | (MH "Professional-Patient Relations") OR (MH "Professional-Family Relations") OR (MH "Professional-Client Relations") | Expanders - Apply related words; Apply equivalent subjects  Search modes - Boolean/Phrase | 59,437 |
| S76 | TI ( (continuit* or continuum or path or paths or pathway* or transition*) W0 (care or "health care" or "health-care" or healthcare) ) OR AB ( (continuit* or continuum or path or paths or pathway* or transition*) W0 (care or "health care" or "health-care" or healthcare) ) | Expanders - Apply related words; Apply equivalent subjects  Search modes - Boolean/Phrase | 1,960 |
| S75 | (MH "Transitional Care") | Expanders - Apply related words; Apply equivalent subjects  Search modes - Boolean/Phrase | 3,022 |
| S74 | (MH "Transfer, Discharge") | Expanders - Apply related words; Apply equivalent subjects  Search modes - Boolean/Phrase | 6,396 |
| S73 | (MH "Continuity of Patient Care+") | Expanders - Apply related words; Apply equivalent subjects  Search modes - Boolean/Phrase | 21,014 |
| S72 | TI ( (client* or patient or patients or facility or facilities or hospital or hospitals) N3 discharg* ) OR AB ( (client* or patient or patients or facility or facilities or hospital or hospitals) N3 discharg* ) | Expanders - Apply related words; Apply equivalent subjects  Search modes - Boolean/Phrase | 41,592 |
| S71 | (MH "Patient Discharge") OR (MH "Discharge Planning+") | Expanders - Apply related words; Apply equivalent subjects  Search modes - Boolean/Phrase | 26,081 |
| S70 | TI ( (after or followup or "follow-up") W0 (care or hospital* or treatment) ) OR AB ( (after or followup or "follow-up") W0 (care or hospital* or treatment) ) | Expanders - Apply related words; Apply equivalent subjects  Search modes - Boolean/Phrase | 37,584 |
| S69 | TI aftercare OR AB aftercare | Expanders - Apply related words; Apply equivalent subjects  Search modes - Boolean/Phrase | 1,389 |
| S68 | (MH "After Care") | Expanders - Apply related words; Apply equivalent subjects  Search modes - Boolean/Phrase | 17,910 |
| S67 | TI ( (person* or self) n3 (manag* or care) ) OR AB ( (person* or self) n3 (manag* or care) ) | Expanders - Apply related words; Apply equivalent subjects  Search modes - Boolean/Phrase | 63,305 |
| S66 | (MH "Self-Management") | Expanders - Apply related words; Apply equivalent subjects  Search modes - Boolean/Phrase | 2,032 |
| S65 | (MH "Self Care") | Expanders - Apply related words; Apply equivalent subjects  Search modes - Boolean/Phrase | 43,441 |
| S64 | TI ( (caregiver* or (care W0 giver#) or client* or family or families or patient* or person or persons or personal or personally or user#) N3 (activat* or engag* or empower* or involv* or participat*) ) OR AB ( (caregiver* or (care W0 giver#) or client* or family or families or patient* or person or persons or personal or personally or user#) N3 (activat* or engag* or empower* or involv* or participat*) ) | Expanders - Apply related words; Apply equivalent subjects  Search modes - Boolean/Phrase | 87,843 |
| S63 | (MH "Consumer Participation") | Expanders - Apply related words; Apply equivalent subjects  Search modes - Boolean/Phrase | 22,735 |
| S62 | TI ( ("client-centered" or "client-centred" or (client W0 focus#ed)) N3 (approach or approaches or care or healthcare or "health care" or model or models or rehab*) ) OR AB ( ("client-centered" or "client-centred" or (client W0 focus#ed)) N3 (approach or approaches or care or healthcare or "health care" or model or models or rehab*) ) | Expanders - Apply related words; Apply equivalent subjects  Search modes - Boolean/Phrase | 844 |
| S61 | TI ( ("patient-centered" or "patient-centred" or (patient W0 focus#ed)) N3 (approach or approaches or care or healthcare or "health care" or model or models or rehab*) ) OR AB ( ("patient-centered" or "patient-centred" or (patient W0 focus#ed)) N3 (approach or approaches or care or healthcare or "health care" or model or models or rehab*) ) | Expanders - Apply related words; Apply equivalent subjects  Search modes - Boolean/Phrase | 9,861 |
| S60 | (MH "Patient Centered Care") | Expanders - Apply related words; Apply equivalent subjects  Search modes - Boolean/Phrase | 33,765 |
| S59 | TI ( (caregiver# or (care W0 giver#) or client# or family or families or patient# or person or persons or personal or personally or user#) N3 (communicat* or educat* or inform* or instruct* or resourc* or teach*) ) OR AB ( (caregiver# or (care W0 giver#) or client# or family or families or patient# or person or persons or personal or personally or user#) N3 (communicat* or educat* or inform* or instruct* or resourc* or teach*) ) | Expanders - Apply related words; Apply equivalent subjects  Search modes - Boolean/Phrase | 137,322 |
| S58 | (MH "Patient Education") OR (MH "Patient Discharge Education") | Expanders - Apply related words; Apply equivalent subjects  Search modes - Boolean/Phrase | 71,176 |
| S57 | TI ( (care or "health care" or "health-care" or healthcare) W0 (educat* or communicat* or inform* or instruct*) ) AND AB ( (care or "health care" or "health-care" or healthcare) W0 (educat* or communicat* or inform* or instruct*) ) | Expanders - Apply related words; Apply equivalent subjects  Search modes - Boolean/Phrase | 714 |
| S56 | TI ( (communicat* or provid* or provision* or seek* or search* or shar* or sought) N3 information ) OR AB ( seek* or search* or shar* or sought) N3 information ) | Expanders - Apply related words; Apply equivalent subjects  Search modes - Boolean/Phrase | 17,714 |
| S55 | (MH "Information Seeking Behavior") | Expanders - Apply related words; Apply equivalent subjects  Search modes - Boolean/Phrase | 4,754 |
| S54 | TI ( misinform* or (mis W0 inform*) ) OR AB ( misinform* or (mis W0 inform*) ) | Expanders - Apply related words; Apply equivalent subjects  Search modes - Boolean/Phrase | 2,301 |
| S53 | TI ( misunderstand* or (mis W0 understand*) ) OR AB ( misunderstand* or (mis W0 understand*) ) | Expanders - Apply related words; Apply equivalent subjects  Search modes - Boolean/Phrase | 2,836 |
| S52 | TI ( miscommunicat* or (mis W0 communicat*) ) OR AB ( miscommunicat* or (mis W0 communicat*) ) | Expanders - Apply related words; Apply equivalent subjects  Search modes - Boolean/Phrase | 656 |
| S51 | TI communicat* | Expanders - Apply related words; Apply equivalent subjects  Search modes - Boolean/Phrase | 38,879 |
| S50 | (MH "Communication") | Expanders - Apply related words; Apply equivalent subjects  Search modes - Boolean/Phrase | 87,833 |
| S49 | TI ( outpatient* or (out W0 patient*) ) OR AB ( outpatient* or (out W0 patient*) ) | Expanders - Apply related words; Apply equivalent subjects  Search modes - Boolean/Phrase | 74,194 |
| S48 | (MH "Surgical Patients") | Expanders - Apply related words; Apply equivalent subjects  Search modes - Boolean/Phrase | 11,545 |
| S47 | (MH "Outpatients") | Expanders - Apply related words; Apply equivalent subjects  Search modes - Boolean/Phrase | 49,228 |
| S46 | (MH "Rehabilitation Patients") | Expanders - Apply related words; Apply equivalent subjects  Search modes - Boolean/Phrase | 3,482 |
| S45 | (MH "Inpatients") | Expanders - Apply related words; Apply equivalent subjects  Search modes - Boolean/Phrase | 85,334 |
| S44 | (MH "Patients") | Expanders - Apply related words; Apply equivalent subjects  Search modes - Boolean/Phrase | 9,524 |
| S43 | TI ( ambulatory N3 (care or "health care" or "health-care" or healthcare) ) OR AB ( ambulatory N3 (care or "health care" or "health-care" or healthcare) ) | Expanders - Apply related words; Apply equivalent subjects  Search modes - Boolean/Phrase | 6,929 |
| S42 | (MH "Ambulatory Care") | Expanders - Apply related words; Apply equivalent subjects  Search modes - Boolean/Phrase | 13,100 |
| S41 | (MH "Family") | Expanders - Apply related words; Apply equivalent subjects  Search modes - Boolean/Phrase | 45,019 |
| S40 | (MH "Caregivers") | Expanders - Apply related words; Apply equivalent subjects  Search modes - Boolean/Phrase | 40,218 |
| S39 | TI rehab* | Expanders - Apply related words; Apply equivalent subjects  Search modes - Boolean/Phrase | 47,368 |
| S38 | TI ( (exercis* or remedial* or rehab*) N3 therap* ) OR AB ( (exercis* or remedial* or rehab*) N3 therap* ) | Expanders - Apply related words; Apply equivalent subjects  Search modes - Boolean/Phrase | 11,547 |
| S37 | (MH "Therapeutic Exercise") | Expanders - Apply related words; Apply equivalent subjects  Search modes - Boolean/Phrase | 26,118 |
| S36 | (MH "Rehabilitation Nursing") | Expanders - Apply related words; Apply equivalent subjects  Search modes - Boolean/Phrase | 1,862 |
| S35 | (MH "Telerehabilitation") | Expanders - Apply related words; Apply equivalent subjects  Search modes - Boolean/Phrase | 432 |
| S34 | (MH "Rehabilitation") | Expanders - Apply related words; Apply equivalent subjects  Search modes - Boolean/Phrase | 17,328 |
| S33 | TI ( ((after W0 operati*) or (after W0 surger*) or postoperati* or (post W0 operati*) or postsurg* or (post W0 surg*)) N3 (care or "health care" or "health-care" or healthcare or followup or "follow-up" or rehab* or therap* or treatment*) ) OR AB ( ((after W0 operati*) or (after W0 surger*) or postoperati* or (post W0 operati*) or postsurg* or (post W0 surg*)) N3 (care or "health care" or "health-care" or healthcare or followup or "follow-up" or rehab* or therap* or treatment*) ) | Expanders - Apply related words; Apply equivalent subjects  Search modes - Boolean/Phrase | 20,075 |
| S32 | TI ( (geriatric* or home) N3 (care or "health care" or "health-care" or healthcare) ) OR AB ( (geriatric* or home) N3 (care or "health care" or "health-care" or healthcare) ) | Expanders - Apply related words; Apply equivalent subjects  Search modes - Boolean/Phrase | 40,811 |
| S31 | (MH "Home Nursing") OR (MH "Home Health Care") OR (MH "Home Rehabilitation+") | Expanders - Apply related words; Apply equivalent subjects  Search modes - Boolean/Phrase | 30,406 |
| S30 | (MH "Gerontologic Nursing") | Expanders - Apply related words; Apply equivalent subjects  Search modes - Boolean/Phrase | 13,571 |
| S29 | TI healthservice* or (health W0 service*) or (healthcare W0 service#) or ("health care" W0 service#) | Expanders - Apply related words; Apply equivalent subjects  Search modes - Boolean/Phrase | 18,790 |
| S28 | (MH "Urban Health Services") | Expanders - Apply related words; Apply equivalent subjects  Search modes - Boolean/Phrase | 1,397 |
| S27 | (MH "Rural Health Services") | Expanders - Apply related words; Apply equivalent subjects  Search modes - Boolean/Phrase | 7,305 |
| S26 | (MH "Community Health Nursing") | Expanders - Apply related words; Apply equivalent subjects  Search modes - Boolean/Phrase | 28,412 |
| S25 | (MH "Community Health Services") | Expanders - Apply related words; Apply equivalent subjects  Search modes - Boolean/Phrase | 23,607 |
| S24 | (MH "Health Services") | Expanders - Apply related words; Apply equivalent subjects  Search modes - Boolean/Phrase | 14,916 |
| S23 | TI (deliver* or model or models or provid* or provision) N3 (care or "health care" or "health-care" or healthcare) | Expanders - Apply related words; Apply equivalent subjects  Search modes - Boolean/Phrase | 27,973 |
| S22 | (MH "Health Care Delivery, Integrated") | Expanders - Apply related words; Apply equivalent subjects  Search modes - Boolean/Phrase | 13,557 |
| S21 | (MH "Health Care Delivery") | Expanders - Apply related words; Apply equivalent subjects  Search modes - Boolean/Phrase | 60,046 |
| S20 | (MH "Hip Fractures+/PC/RH/SU/TH") | Expanders - Apply related words; Apply equivalent subjects  Search modes - Boolean/Phrase | 7,300 |
| S19 | S4 OR S5 OR S6 OR S7 OR S8 OR S9 OR S10 OR S11 OR S12 OR S13 OR S14 OR S15 OR S16 OR S17 OR S18 | Expanders - Apply related words; Apply equivalent subjects  Search modes - Boolean/Phrase | 396,682 |
| S18 | TI internet* or app or apps or computer* or cyber* or (e W0 application#) or (e W0 mail*) or email* or (electronic W0 mail*) or iphone# or (i W0 phone#) or (mobile N2 application#) or "mobile-based" or (mobile W0 phone#) or online or (patient# N3 portal*) or (instant* W0 messag*) or (secure* N3 communicat*) or (secure* N3 messag*) or (secure* N3 platform*) or (secure* N3 portal*) or service or services or smarthome* or (smart W0 home*) or smarthub# or (smart W0 hub#) or smartphone* or (smart W0 phone*) or technolog* or telecommunicat* or (tele W0 communicat*) or telephon* or textmessag* or (text W0 messag*) or video* or web or webbased or "web-based" or webdeliver* or (web W0 deliver*) or "web 2.0" | Expanders - Apply related words; Apply equivalent subjects  Search modes - Boolean/Phrase | 250,863 |
| S17 | TI ( (internet* or app or apps or computer* or cyber* or (e W0 application#) or (e W0 mail*) or email* or (electronic W0 mail*) or iphone# or (i W0 phone#) or (mobile N2 application#) or "mobile-based" or (mobile W0 phone#) or online or (patient# N3 portal*) or (instant* W0 messag*) or (secure* N3 communicat*) or (secure* N3 messag*) or (secure* N3 platform*) or (secure* N3 portal*) or service or services or smarthome* or (smart W0 home*) or smarthub# or (smart W0 hub#) or smartphone* or (smart W0 phone*) or technolog* or telecommunicat* or (tele W0 communicat*) or telephon* or textmessag* or (text W0 messag*) or video* or web or webbased or "web-based" or webdeliver* or (web W0 deliver*) or "web 2.0") N3 (care or "health care" or "health-care" or healthcare or appointment* or coach* or conferenc* or consult* or interven* or manag* or meet* or monitor* or rehab* or support* or therap* or treatment# or visit*) ) OR AB ( (internet* or app or apps or computer* or cyber* or (e W0 application#) or (e W0 mail*) or email* or (electronic W0 mail*) or iphone# or (i W0 phone#) or (mobile N2 application#) or "mobile-based" or (mobile W0 phone#) or online or (patient# N3 portal*) or (instant* W0 messag*) or (secure* N3 communicat*) or (secure* N3 messag*) or (secure* N3 platform*) or (secure* N3 portal*) or service or services or smarthome* or (smart W0 home*) or smarthub# or (smart W0 hub#) or smartphone* or (smart W0 phone*) or technolog* or telecommunicat* or (tele W0 communicat*) or telephon* or textmessag* or (text W0 messag*) or video* or web or webbased or "web-based" or webdeliver* or (web W0 deliver*) or "web 2.0") N3 (care or "health care" or "health-care" or healthcare or appointment* or coach* or conferenc* or consult* or interven* or manag* or meet* or monitor* or rehab* or support* or therap* or treatment# or visit*) ) | Expanders - Apply related words; Apply equivalent subjects  Search modes - Boolean/Phrase | 160,880 |
| S16 | (MH "Therapy, Computer Assisted") | Expanders - Apply related words; Apply equivalent subjects  Search modes - Boolean/Phrase | 5,478 |
| S15 | (MH "Internet-Based Intervention") | Expanders - Apply related words; Apply equivalent subjects  Search modes - Boolean/Phrase | 409 |
| S14 | TI ( mobile W0 (provider# or clinician# or counsel#or# or doctor# or nurse# or physician# or practitioner# or therapist#) ) OR AB ( mobile W0 (provider# or clinician# or counsel#or# or doctor# or nurse# or physician# or practitioner# or therapist#) ) | Expanders - Apply related words; Apply equivalent subjects  Search modes - Boolean/Phrase | 49 |
| S13 | TI ( e-provider# or e-clinician# or e-counsel#or# or e-doctor# or e-nurse# or e-physician# or e-practitioner# or e-therapist# or m-provider# or m-clinician# or m-counsel#or# or m-doctor# or m-nurse# or m-physician# or m-practitioner# or m-therapist# ) OR AB ( e-provider# or e-clinician# or e-counsel#or# or e-doctor# or e-nurse# or e-physician# or e-practitioner# or e-therapist# or m-provider# or m-clinician# or m-counsel#or# or m-doctor# or m-nurse# or m-physician# or m-practitioner# or m-therapist# ) | Expanders - Apply related words; Apply equivalent subjects  Search modes - Boolean/Phrase | 350 |
| S12 | TI ( (digital* or virtual* or remote*) N3 (appointment* or clinic or clinics or coach* or communicat* or conferenc* or consult* or followup or "follow-up" or hub or hubs or interven* or manag* or meet* or monitor* or rehab* or support* or therap* or tool or tools or treatment# or visit*) ) OR AB ( (digital* or virtual* or remote*) N3 (appointment* or clinic or clinics or coach* or communicat* or conferenc* or consult* or followup or "follow-up" or hub or hubs or interven* or manag* or meet* or monitor* or rehab* or support* or therap* or tool or tools or treatment# or visit*) ) | Expanders - Apply related words; Apply equivalent subjects  Search modes - Boolean/Phrase | 21,849 |
| S11 | TI ( (digital* or virtual* or remote*) N3 (care or health* or healthcare or "health-care") ) OR AB ( (digital* or virtual* or remote*) N3 (care or health* or healthcare or "health-care") ) | Expanders - Apply related words; Apply equivalent subjects  Search modes - Boolean/Phrase | 10,777 |
| S10 | TI ( mobile W0 (health* or care or counsel* or medicine or psychiatr* or psycholog*) ) OR AB ( mobile W0 (health* or care or counsel* or medicine or psychiatr* or psycholog*) ) | Expanders - Apply related words; Apply equivalent subjects  Search modes - Boolean/Phrase | 2,660 |
| S9 | TI ( emedicine* or e-medicine* ) OR AB ( emedicine* or e-medicine* ) | Expanders - Apply related words; Apply equivalent subjects  Search modes - Boolean/Phrase | 58 |
| S8 | TI ( ecoach* or e-coach* ) OR AB ( ecoach* or e-coach* ) | Expanders - Apply related words; Apply equivalent subjects  Search modes - Boolean/Phrase | 39 |
| S7 | TI ( ehealth* or e-health* or mhealth* or m-health* or emental health* or e-mental health* or mmental health* or m-mental health* or epsychiatr* or e-psychiatr* or mpsychiatr* or m-psychiatr* or epsychol* or e-psychol* or mpsychol* or m-psychol* or erehab* or e-rehab* or mrehab* or m-rehab* or etherap* or e-therap* or esupport* or e-support* or msupport* or m-support* or evisit* or e-visit* or mvisit* or m-visit* ) OR AB ( ehealth* or e-health* or mhealth* or m-health* or emental health* or e-mental health* or mmental health* or m-mental health* or epsychiatr* or e-psychiatr* or mpsychiatr* or m-psychiatr* or epsychol* or e-psychol* or mpsychol* or m-psychol* or erehab* or e-rehab* or mrehab* or m-rehab* or etherap* or e-therap* or esupport* or e-support* or msupport* or m-support* or evisit* or e-visit* or mvisit* or m-visit* ) | Expanders - Apply related words; Apply equivalent subjects  Search modes - Boolean/Phrase | 7,210 |
| S6 | TI ( telemed* or tele-med* or telecare or tele-care or teleconsult* or tele-consult* or teleconferenc* or tele-conferenc* or telecounsel* or tele-counsel* or telehealth* or tele-health* or telemonitor* or tele-monitor* or telepsychiatr* or tele-psychiatr* or telepsycholog* or tele-psycholog* or telerehab* or tele-rehab* or telesupport* or tele-support* or teletherap* or tele-therap* or teletreatment* or tele-treatment* ) OR AB ( telemed* or tele-med* or telecare or tele-care or teleconsult* or tele-consult* or teleconferenc* or tele-conferenc* or telecounsel* or tele-counsel* or telehealth* or tele-health* or telemonitor* or tele-monitor* or telepsychiatr* or tele-psychiatr* or telepsycholog* or tele-psycholog* or telerehab* or tele-rehab* or telesupport* or tele-support* or teletherap* or tele-therap* or teletreatment* or tele-treatment* ) | Expanders - Apply related words; Apply equivalent subjects  Search modes - Boolean/Phrase | 17,131 |
| S5 | (MH "Telenursing") OR (MH "Telehealth") | Expanders - Apply related words; Apply equivalent subjects  Search modes - Boolean/Phrase | 14,119 |
| S4 | (MH "Telemedicine+") | Expanders - Apply related words; Apply equivalent subjects  Search modes - Boolean/Phrase | 18,128 |
| S3 | S1 OR S2 | Expanders - Apply related words; Apply equivalent subjects  Search modes - Boolean/Phrase | 17,960 |
| S2 | TI ( (hip or hips or femoral neck or (femur# N2 neck#) or acetabul* or intertrochanter* or inter-trochanter* or pertrochanter* or per-trochanter* or subtrochanter* or sub-trochanter* or trochanter*) N3 (break* or broke* or fractur*) ) OR AB ( (hip or hips or femoral neck or (femur# N2 neck#) or acetabul* or intertrochanter* or inter-trochanter* or pertrochanter* or per-trochanter* or subtrochanter* or sub-trochanter* or trochanter*) N3 (break* or broke* or fractur*) ) | Expanders - Apply related words; Apply equivalent subjects  Search modes - Boolean/Phrase | 15,676 |
| S1 | (MH "Hip Fractures+") | Expanders - Apply related words; Apply equivalent subjects  Search modes - Boolean/Phrase | 11,831 |
